# HIV-1 lentivirus with VSV-G envelope: Complex N glycosylation governs shifts in aggregation and infectivity state at close to physiological pH, serum, and Ca^2+^ levels

**DOI:** 10.1371/journal.pone.0357567

**Published:** 2026-09-09

**Authors:** Tzu-Lan Chang, Ayobami I. Ogundiran, Andrey Ivanov, Gabrielle Torain, Sergei Nekhai, Preethi L. Chandran

**Affiliations:** 1 Department of Chemical Engineering, Howard University, Washington, District of Columbia, United States of America; 2 Center for Sickle Cell Disease, Howard University, Washington, District of Columbia, United States of America; Emory University, UNITED STATES OF AMERICA

## Abstract

The goal of the study is to determine if the N-glycosylation on a virus affects its diffusion or solution state in different environmental conditions, and if the nature of the solution states has any consequence for the virus infectivity. HIV-1 lentivirus is frequently pseudo-typed with the envelope protein of Vesicular Stomatitis Virus, VSV-G, for gene and cell therapy applications. VSV-G proteins have complex type N-glycans. The diffusion profile of the lentivirus having VSV-G envelope proteins was monitored in different solution conditions using Dynamic Light Scattering, and inferences checked by AFM, filtration, antibody, and p24 ELISA experiments. The virus switched between one of three solution states in response to changes in its environment: (a) a self-aggregated state when serum to virus ratio was low, and Ca^2+^ and pH levels were below 2 mM and 7.4, respectively, (b) a dispersed state when serum to virus ratio was high, but Ca^2+^ and pH levels were still below 2mM and 7.4, respectively, and (c) a Ca-induced aggregated state when Ca^2+^ and pH levels exceeded 2mM and 7.4 respectively. The diffusion peak of the aggregated virus can be pulled out by antibodies against VSV-G or can be filtered out with attendant loss in p24 count and infectivity. The three solution states exhibited significantly different infectivity levels. Interestingly, the trigger conditions for switching the virus solution state occur at physiological levels of pH, Ca^2+^, and serum, implying that small deviations from the homeostatic conditions can induce large changes in virus infectivity. The ability to exist and switch between three solution states was lost when the terminal sialic acid and galactose residues on the virus envelope protein were cleaved. It appears that the complex N-glycans on a virus allow the latter to respond to deviations from homeostatic environmental conditions by switching solution states.

## Introduction

**Fig 1:**
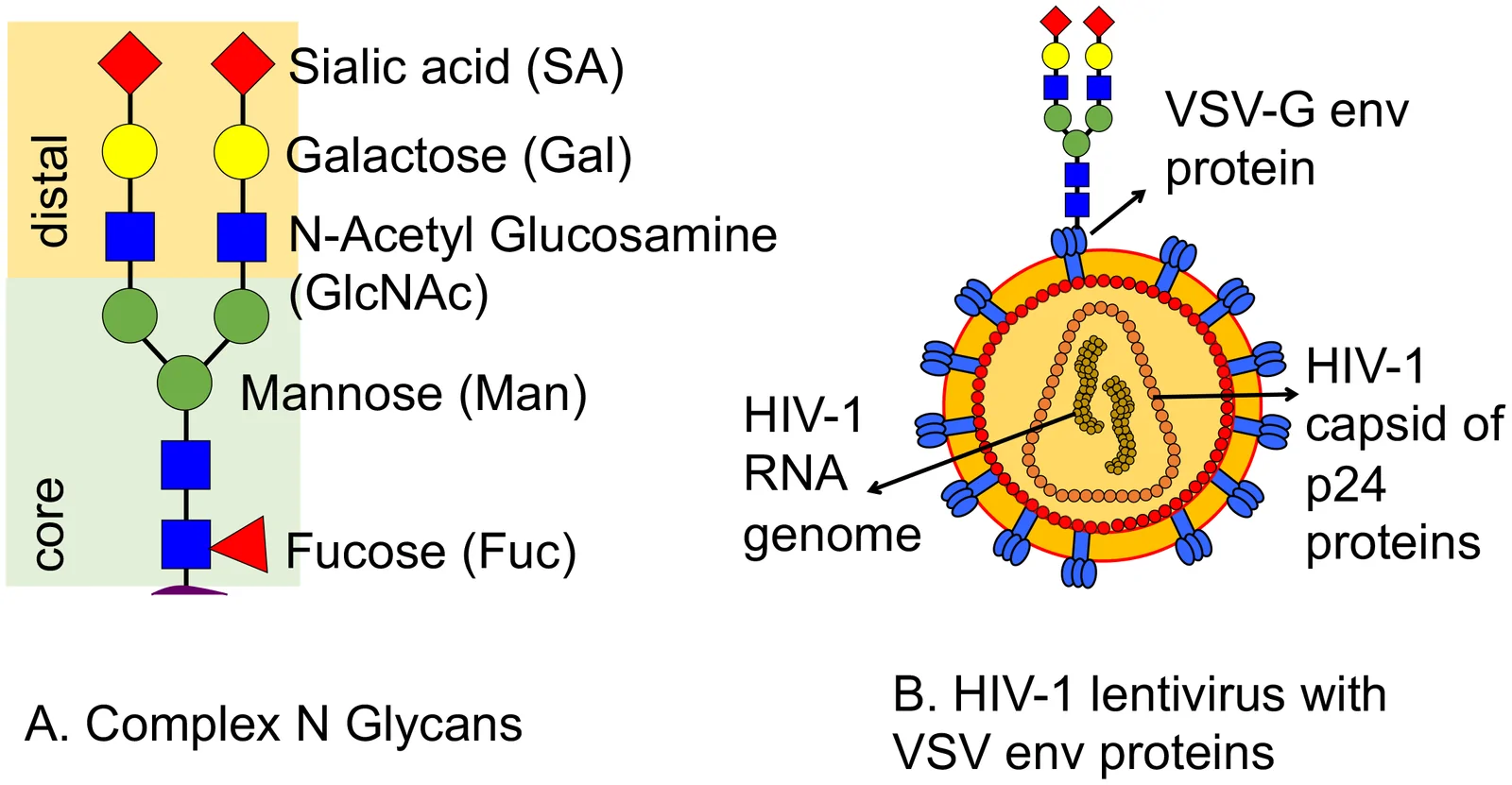
[A] Sequence of sugar residues in complex N glycans, and [B] schematic of HIV-1 viral particle pseudo-typed with VSV-G envelope proteins presenting complex N-glycans (B).

Eukaryotic cells and viruses are covered with branched polymers of sugar residues known as glycans, attached to proteins and lipids in their membrane [1–3]. Greater than 70% of these glycans are of the N-glycan type which attach to proteins via the latter’s amine side groups [4]. N-glycans have a highly conserved sequence of sugars that includes a universal core of N-acetyl glucosamine (GlcNAc) and mannose residues (Fig 1A). N-glycans are classified into three types depending on the residues present distal to the core. The high-mannose type has distal mannose residues, the complex-type has distal GlcNAc and galactose (gal) residues that may be capped with sialic acid (SA) (shown in Fig 1A), and the hybrid type has both high-mannose and complex type branches [5–7]. Glycans constitute the outermost interface of biological units, but little is known if the glycan type presented systematically influences how biological units sample and interact with their environment.

Membrane viruses are useful for understanding the passive environmental sensing and response conferred by glycosylation. They lack internal signaling mechanisms to actively sense and respond to the environment. In this work, we determine if glycosylation influences how HIV-1 lentivirus with VSV-G envelope proteins having complex N-glycans (Fig 1B) presents itself in solution in environmental conditions that are well within the range of the virus culture and working conditions, and if changes in the solution states have consequences for virus virulence. The premise of our work comes from earlier observations where the surface adhesion biophysics of VSV-G env lentivirus was found to change from Slime-like to Velcro-like to Teflon-like as the SA, mannose, and GlcNAc sugars on the virus glycans were sequentially exposed (see Fig 1A). While changes in the surface glycosylation of the lentivirus produced large changes in its surface interactions, little is known if it would translate to solution level behavior. We had reported the solution behavior of HIV-1 lentivirus having gp160 envelope proteins with high mannose N-glycans. The virus occurred self-aggregated in culture media, the aggregation not affected by serum or salt levels, but reversibly dispersed by filtration shear, and is lost when the mannose residues were cleaved. The brittle Velcro-like surface adhesion observed between lentivirus presenting terminal mannose residues translated in solution to a brittle self-aggregation that allowed aggregates to pass through filter membranes and reaggregate on the other side. It is not known if lentivirus presenting complex N-glycans with terminal SA residues would exhibit similar or different solution behavior.

In this study we examine the diffusion states adopted by HIV-1 lentivirus having envelope glycoproteins or env from Vesicular Stomatitis Virus (i.e., VSV-G) (Fig 1B). The VSV-G env display complex N-glycans irrespective of the host cell used for production. The presence of these glycans is critical for the folding and trafficking of the envelope protein within host cells and for their packaging into budding virus [8–11]. HIV-1 lentivirus carrying VSV-G env have been widely used in gene- and cell- therapies because of the ubiquity of the LDL receptors that the VSV-G env binds to [12–17]. We investigate if a lentivirus covered with complex N-glycans assumes consistent solution states in response to specific environmental triggers, if these diffusion states are different than those adopted by high-mannose lentivirus, if the diffusion states are lost when the virus N-glycans are trimmed, and if the diffusion states bear any consequence to the virus infectivity.

Dynamic Light Scattering (DLS) was used to monitor the changing landscape of diffusion sizes as lentiviruses aggregated, dispersed, and interacted with proteins in solution; with other techniques providing orthogonal confirmation. Due to the multifold difference in the size of free serum proteins (~10–20 nm), serum aggregates (~60nm) and free virus particles (~60–100 nm), and virus aggregates (>200 nm), the exchange between free and aggregated virus and proteins can be monitored with the size resolution afforded by DLS. Lentivirus with VSV-G env having complex N-glycans exhibited solution behavior (i.e., solution or diffusion states in different environmental conditions) that was diametrically opposite to lentivirus having gp160 env and presenting high-mannose glycans. Well-defined and reproducible changes in the diffusion states of VSV-G env lentivirus were triggered by minor drifts from homeostatic conditions of pH, Ca^2+^, and serum; and changes in the diffusion states produced multifold changes in the infectivity of the virus in cell culture.

## Materials and methods

### Preparation of HIV-1 lentivirus with VSV-G env

HEK 293T cells were purchased from ATCC (Manassas, VA). The following reagent was obtained through the NIH HIV Reagent Program, Division of AIDS, NIAID, NIH: Human Immunodeficiency Virus Type 1 (HIV-1) NL4–3 Δ*Env Vpr* Luciferase Reporter Vector (pNL4–3.Luc.R-E-), ARP-3418, contributed by Dr. Nathaniel Landau.[18,19] The HIV-1 proviral DNA pNL4–3.Luc.R-E (HIV-Luc-G) plasmid contains two nonsense frame shifts in the *env* and *vpr* genes, and a Luciferase reporter gene cloned in place of *nef*. The plasmid pHEF VSVg expressing the envelope protein of Vesicular Stomatitis Virus (VSV-G) was also obtained from NIH AIDS Reagent Program (Germantown, MD). For cell culture, DMEM (Dulbecco’s Modified Eagle Medium, high glucose, Gibco^TM^, Catalog number: 11965118) was supplemented with 10% FBS (Gibco™ to, 26140087). For virus production HEK cells were grown 70–90% confluency in 6 well plates. DNA transfection particles were prepared using Lipofectamine™ 3000 (Invitrogen™) and Opti-MEM^®^ according to manufacturer protocol to deliver 3µg DNA per well of envelope and provirus plasmids in the ratio of 1:4. The cells were incubated for 2–4 days at 37°C before the culture supernatant was harvested for virus collection.[20] The virus containing culture media was clarified of debris with 3,000xg centrifugation for 15 min and stored long-term at −80°C in aliquots. This preparation is referred to as virus media. Viral titer was estimated to be 2x10^5^ – 20x10^5^ transducing viral particles /ml based on the p24 concentration (500 ng/ml) determined by Takara ELISA assay (TakaraBio). p24 count of bald virus in media was about 20 ng/ml. Virus was diluted as needed for experiments in either phosphate buffered saline (PBS), pH 7.4 (Caisson Labs, PBL06, 4 mM, Smithfield, Utah), or PBS titrated with HCl (Sigma, H9892) to achieve pH of 6.5, or with DMEM without or with the addition of 10% FBS. For imaging experiment, virus without serum interference, 10 ml of clarified media containing virus was further ultracentrifuged to 35,000 RPM (Beckman/Coulter Optima XPN-90) for 2 hours at 4°C and the pellet was resuspended in 100 µl of PBS. DLS signal and p24 assay following several orders dilution of the suspension indicated ~100 X concentrated virus. Control media was also subjected to similar treatment.

### Glycosidases, lectins, and antibodies

Terminal sialic acid (SA) and galactose (gal) residues on the virus glycans were cleaved with glycosidases Neuraminidase (NA) from *Vibrio cholera* (Roche,11080725001) and β-Galactosidase (β-Gal) from *E. coli* (Roche,10105031001), respectively. The solutions of glycosidases were added directly in the following volumes: 5 μL for NA, 2 μL for β-Gal, and 5 μL + 2 μL for NA + β-Gal mixture, to a final volume of 45 μL of virus-media (9X diluted in PBS). The solution was incubated for two hours at 37°C for cleavage activity. The 1 mg/ml stock of NA and β-Gal were added at 5 µL and 2 µL volume, respectively, to virus-media 9X diluted in PBS to always get a 45 µL final volume for DLS measurements. Incubation was for two hours at 37°C. The lectins wheat germ agglutinin (WGA, Sigma, L9640) or Erythrina cristagalli agglutinin (ECA, GlycoMatrix, 21510011) were used to identify terminal GlcNAc/SA and gal residues on the virus glycans, respectively. Lectins were reconstituted to 1 mg/mL stock concentration in PBS, aliquoted, and stored at −20°C. The lectin working concentration was maintained at 100 µg/mL. The incubation time for the lectin study was one hour at 25°C. Antibodies to gp120 and VSV-G envelope proteins were purchased from Abcam (Waltham, MA, USA, ab21179) and ThermoFisher Scientific Inc. (Piscataway, NJ, USA, A00199-40), respectively. Diluted virus media was incubated with Abs for one hour at 25°C.

### Dynamic Light Scattering (DLS)

The hydrodynamic diameter (D_H_) or diffusion speed of serum proteins and virus was tracked with DLS to monitor changes in the interaction and aggregation state upon solvent change, and with the addition of serum proteins, lectins, antibodies, etc. Aggregation or coating of the virus is expected to slow its diffusion speed and to be manifested as a rightward shift in the timescale at which the correlation curve decays, and as an increase in hydrodynamic diameter (D_H_). Both correlation curves (unscaled intensity) and D_H_ histograms (scaled intensity) are shown to infer changes in the diffusing species in solution. DLS experiments were performed with 50 µL solution in disposable cuvettes (Malvern, ZEN0040) measured using a Zetasizer Nano ZS (Malvern Panalytical, Inc., Westborough, MA) with 632.8 nm laser wavelength, 173^o^ back-scatter angle, laser attenuation of 9, sampling position of 4.65 mm, equilibration time of 120 sec, and measurement time of 120 sec [21]. The optimal measurement parameters were determined and held constant for all samples, so the correlation curves from different samples can be compared. Particle D_H_ histograms were generated with Zetasizer analysis software v7.11. The contributions to the correlation curve from multiple diffusing species of well-separated sizes were deconvolved using the Sequential Extraction of Late Exponentials (SELE) algorithm [22]. The correlation curves show multiple exponential drops when the diffusing species have significant size differences. For instance, the virus media curves showed contributions from both serum (~10 and 50 nm D_H_) and large virus particles (~500 nm D_H_). The correlation curve of the virus-media can change in shape between different virus production stocks when the serum to virus amounts in solution changes. The relative amount of serum to virus would depend on the virus production efficiency in that batch. For D_H_ to be a reliable indicator of the virus aggregation/coating, the solution needs to be in a concentration range where there are no crowding or long-range interaction effects; that is where the scaled shape of the correlation curves and the D_H_ of the species do not change with concentration. On the other hand, if the virus is too dilute or is also not in a good solvent, aggregation-like shifts in the correlation curve will occur with dilution and this concentration regime is also not reliable for interpretations based on hydrodynamic size [23].

### Antibody pull down in DLS

To verify the presence of the VSV-G envelope proteins in the diffusing populations in DLS, 5 µl of 1 mg/ml anti-VSVG antibodies (Thermofisher, A00199-40) was added to the DLS cuvette containing 44 µl of 5X virus media diluted in PBS. After 1 hour incubation at 25C, 1 µl of 1 micron-sizes magnetic beads coated with protein A/G (Pierce Protein A/G Magnetic Beads, Thermofisher, 88802), and 50 X diluted in PBS from manufacturer concentration, was added to the solution in the DLS cuvette and the mixture incubated at room temperature for 1 hour. Neodymium magnets (Trymag, Amazon) were then placed by the cuvette for 20 minutes to settle the beads. The supernatant was pipetted into another DLS cuvette to determine which diffusion peak was pulled out by the antibodies. Complete removal of the added magnetic beads themselves was confirmed by the loss of their distinct 1 micron diffusion peak from the supernatant solution.

### Atomic force microscopy (AFM)

AFM imaging was performed using Bruker Multimode AFM having Nanoscope-V controller (Bruker Nanosurfaces, Inc., Santa Barbara, CA) in tapping mode with OTespaR3 cantilever (Bruker Nano Inc., Camarillo, CA). 5 µL of sample was allowed to air dry for 12 min on freshly cleaved mica before washing with 50 µL of water twice and drying in a stream of N_2_. Images were flattened and processed with Nanoscope Analysis software (Ver 1.5, Bruker Nanosurfaces, Inc., Santa Barbara, CA).

### Transmission electron microscopy (TEM)

10 μL of virus media was absorbed on formvar, a carbon-coated copper grid for 15 minutes. The excess sample was wicked away and the sample was fixed with 4% glutaraldehyde in 0.12 M sodium cacodylate buffer for 5 minutes. The glutaraldehyde solution was removed with filter paper and the samples were washed 4 times with water. The samples were then stained for 1 min with 1% aqueous uranyl acetate. The grids were dried after wicking away the stain with filter paper. Samples were imaged at 80 KV in a FEI Talos F200X transmission electron microscope (ThermoFisher Scientific, Hillsboro, OR) with Ceta 4M camera.

### Infectivity analysis

350 µl each of the three states of virus was prepared by 5X and 2X dilution in cDMEM (for serum dispersed), DMEM (for aggregated), and cDMEM with 4mM Ca was prepared. HEK293T cells were seeded in a 96-well plate at a density of 3X10^4 cells/well. Twenty-four hours after, cell media was discarded and replaced with 100µl of above virus media containing virus in one of three states and a control of cDMEM only with no virus. Four hours after, additional 100 µl of cDMEM was added to each well. Twenty-four hours after, cells were prepared for luciferase assay (Pierce firefly luciferase flash assay kit, catalogue #16174, Thermofisher Scientific). Cells were lysed for 30 min in a mild speed shaker with an ice coolant underneath the cell culture plate to keep the enzyme (luciferase) activity. 100X enhancer was added to the working solution. The luminescence was read with a microplate reader (Tecan Infinite 200 Pro)

### ELISA assay

Levels of p24 in the virus media was determined by the Lenti-X p24 Rapid Titer ELISA (Takara Bio, San Jose, USA). Serial dilutions of virus filtered though 0.2 µM filter and serial dilutions of the retentate and that of unfiltered virus media were tested per manufacturer instruction using HRP-conjugated anti-p24 antibodies supplied by the ELISA kit. The reactions were stopped based on standards color development and read at 450 nm on Tecan Infinite 200 Pro microplate reader. The p24 values were calculated based on the linear approximation of the standards and the dilution factors.

### Statistical robustness

All DLS experiments were repeated at least twice on different days and at least twice with different viral stock, with three measurements per repeat of the experiment. In many cases, repeats were repeated by different personnel as well, to ensure the rigor and robustness of the data. The DLS data with at least six repeats were compiled and analyzed together to avoid bias in selection of representative data. For AFM images, several areas within a mica disk and at least two separate imaging sessions were performed and only consistently observed results are discussed. Main findings were confirmed with multiple DLS experimental setups, in different solvents, and using different techniques (ELISA, filtration, infectivity, AFM) to confirm robustness. Two-tail student T test was used to determine significance of infectivity results. A p value < 0.01 was considered significant.

## Results

### Aggregates of HIV-1 with VSV-G envelope present in virus media

**Fig 2:**
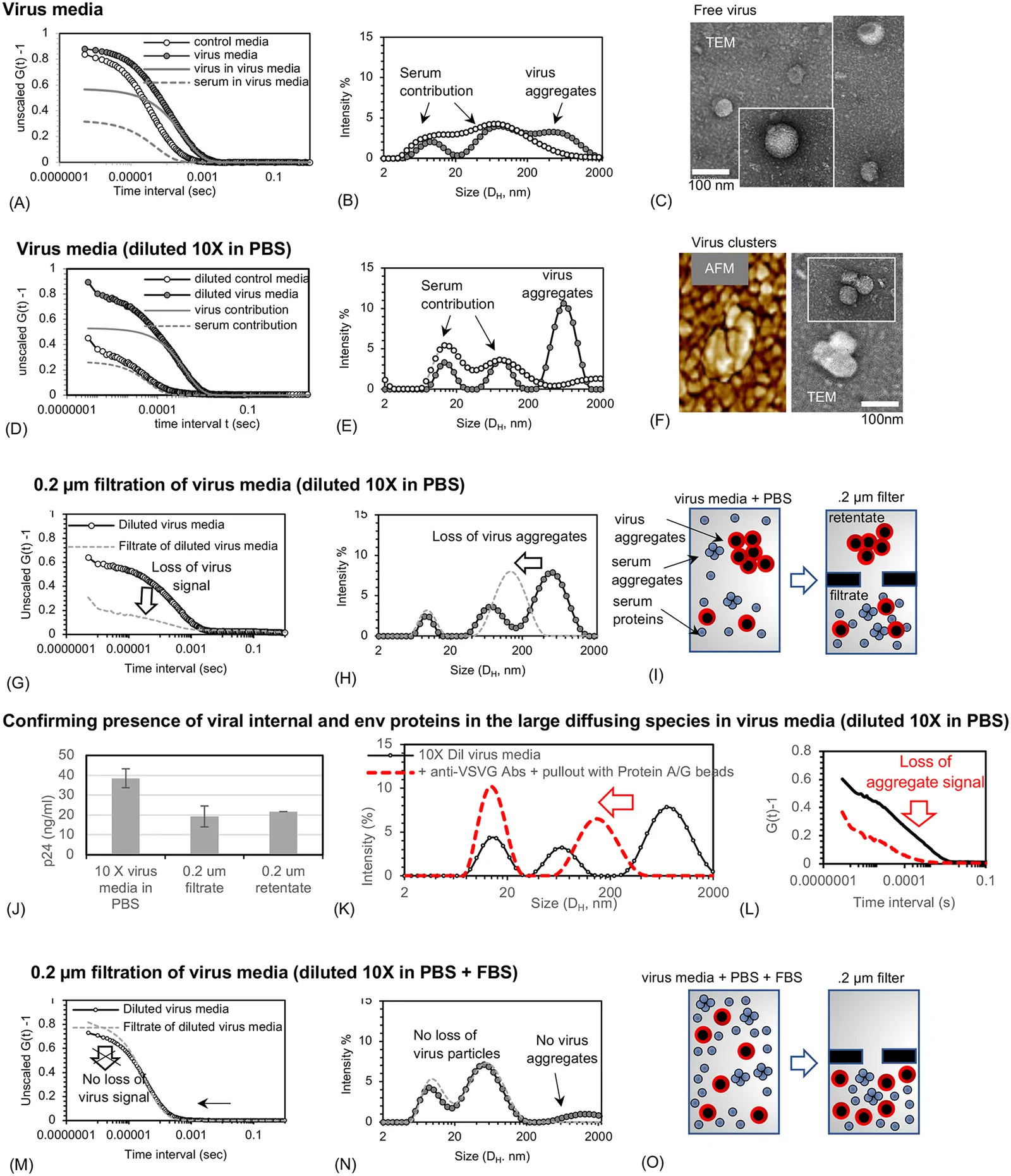
HIV-1 with VSV-G envelope is aggregated in virus media but dispersed by additional Fetal Bovine Serum (FBS): [A] DLS correlation curves of virus in media (culture media containing virus) and control media (culture media alone), with the serum and virus contributions to the virus media curve deconvoluted. [B] Histogram of hydrodynamic diameters (D_H_) showing that both virus media and control media have diffusion peaks corresponding to free (~10 nm D_H_) and aggregated (~60 D_H_) serum proteins, while virus media has an additional ~500 nm D_H_ peak. [C] TEM of virus media showing free virus particles have 60-100 nm diameter. [D] DLS correlation curves of PBS-diluted control and virus media shown along with the deconvoluted serum and virus contributions. [E] DLS histogram corresponding to D. [F] Virus clusters observed in AFM and TEM imaging. [G] DLS curves of PBS-diluted virus media before and after filtration with 0.2 µm filter. [H] DLS histogram corresponding to G. [I] Schematic depicting filter retention of virus-aggregates from virus media. [J] p24 concentration of 10X PBS-diluted virus media and that of the filtrate and retentate phases from  .2µm filtration which were resuspended to have the same volume as the initial virus media. [K] DLS size histogram of 10X PBS-diluted virus media before and after pullout with antibodies against VSV-G. [L] DLS correlation curves corresponding to Fig. 2L. [M] DLS curves of virus-media 10X-diluted with PBS and containing 10% FBS, before and after filtration with 0.2 µm filter. Arrow indicates a leftward shift of the virus media compared to before FBS supplementation. [N] Corresponding histograms showing loss of ~500 nm D_H_ species in FBS supplemented media and no loss of diffusion species when this media is filtered. [O] Schematic showing that addition of FBS appears to disperse the ~ 500 nm D_H_ from virus aggregates, and that dispersed virus pass through a 0.2 µm filter.

Media harvested from HEK 293T cells producing HIV lentivirus with VSV-G env was clarified by centrifugation and referred here as ‘virus media’ to emphasize that its virus and serum contents. Virus media and control media (DMEM + 10% FBS) were examined by DLS. DLS correlation curves provide the autocorrelation between light intensities collected logarithmic time intervals t apart (Fig 2A), at an angle away from the incident light, because of the scattering by particles in solution. The extent of correlation decays exponentially at the time scale of diffusion of the scattering species, and the hydrodynamic diameter (D_H_) of the scattering species is inferred from these diffusion times.

Control media has a strong scattering contribution from serum proteins (Fig 2A), with two diffusing populations observed in the range ~10 and ~60 nm D_H_ (Fig 2B), which are in the size range expected for free proteins and protein aggregates, respectively. Serum proteins in culture media have been reported to aggregate over time to sizes over 25 nm [24]. In addition to the diffusion peaks in control media, virus media has an additional contribution from a ~ 500 nm D_H_ species (Fig 2A, B). Deconvolving the control media contribution (dashed line in Fig 2A) from virus media, for instance, leaves behind a slower decaying correlation curve (solid line in Fig 2A) indicating a larger diffusing species.

The larger diffusing component was absent in media harvested from control cell cultures – that were not transfected, or transfected with lipofectamine alone, or transfected with the plasmid for the envelope protein, or transfected with the plasmid for the bald virus (S1 Fig); which indicated that the large species was likely not cell debris or secretory vesicles which would have featured in the control cultures as well. 10X dilution of virus media with PBS made the ~ 500 nm diffusion peak even more prominent, confirming that it was not a slow diffusing peak because of crowding effects (Fig 2D, E). The 10X dilution was used in subsequent experiments, unless stated otherwise.

The ~ 500 nm D_H_ peak appearing in virus media alone is larger than the 60–100 nm size expected for free lentivirus and observed in AFM and TEM imaging of free virus (Fig 2C). AFM and TEM images of virus media (Fig 2F) showed associative interactions occurring between virus, which were similar despite the two techniques having different sample processing. That these inter-virus associations were invariant to sample-prepping processes suggest that they could be occurring in solution.

Filtration of 10X diluted virus media through 0.2 µm pore size membrane led to removal of the ~ 500 nm D_H_ species. The filtrate had substantially lower scattering intensity (Fig 2G) coming from ~10 nm D_H_ free proteins and the ~ 150 nm D_H_ serum aggregates and free virus (Fig 2H). The retention of the ~ 500 nm D_H_ peak on the filter confirmed it to be a physically large and physically separable entity (Fig 2I). ELISA assay quantified the membrane retentate to contain about half the p24 proteins in virus media, whereas the filtrate contained the other half (n = 5, p = .003) (Fig 2J). Anti-VSVG antibodies completely pulled out the ~500 nm species from 10X diluted virus media, confirming that the ~500 nm diffusing species had VSV-G envelope proteins on the outside (Fig 2K and 2L). There was a partial pullout of the 60-100 nm peak as well, compared to the free-protein peak, suggesting that some virus may be present free in solution.

The DLS and antibody experiments examinations of virus media point to the presence of a large and separable diffusing entity, having VSV-G env proteins on the outside and p24 proteins within, that only appears in the media harvested and clarified from cells producing virus. AFM and TEM images show the presence of free virus of 60–100 nm size and the presence of inter-virus associations. If the 500 nm D_H_ species was an aggregate of smaller virus entities, then some solution condition should be able to trigger its dispersal into the smaller units. The addition of 10% vol/vol FBS to PBS-diluted virus media led to loss of the ~ 500 nm diffusing species and an increase in the relative contribution from the 60–100 nm species in the range of free virus (Fig 2M and 2N). Filtration confirmed the complete dispersal of the large entities into species < 0.2 µm; the filtrate retained all the scattering signal and diffusing species from the dispersed solution (Fig 2M–O). These results together indicate that the ~ 500 nm entities appearing in virus media are aggregates of lentivirus with VSV env.

### Aggregates of HIV-1 with VSV-G envelope dispersed by FBS proteins

The addition of FBS solution dispersed the viral aggregates in virus media. We examined the aspect of the component of the solution dispersing the aggregates.

**Fig 3:**
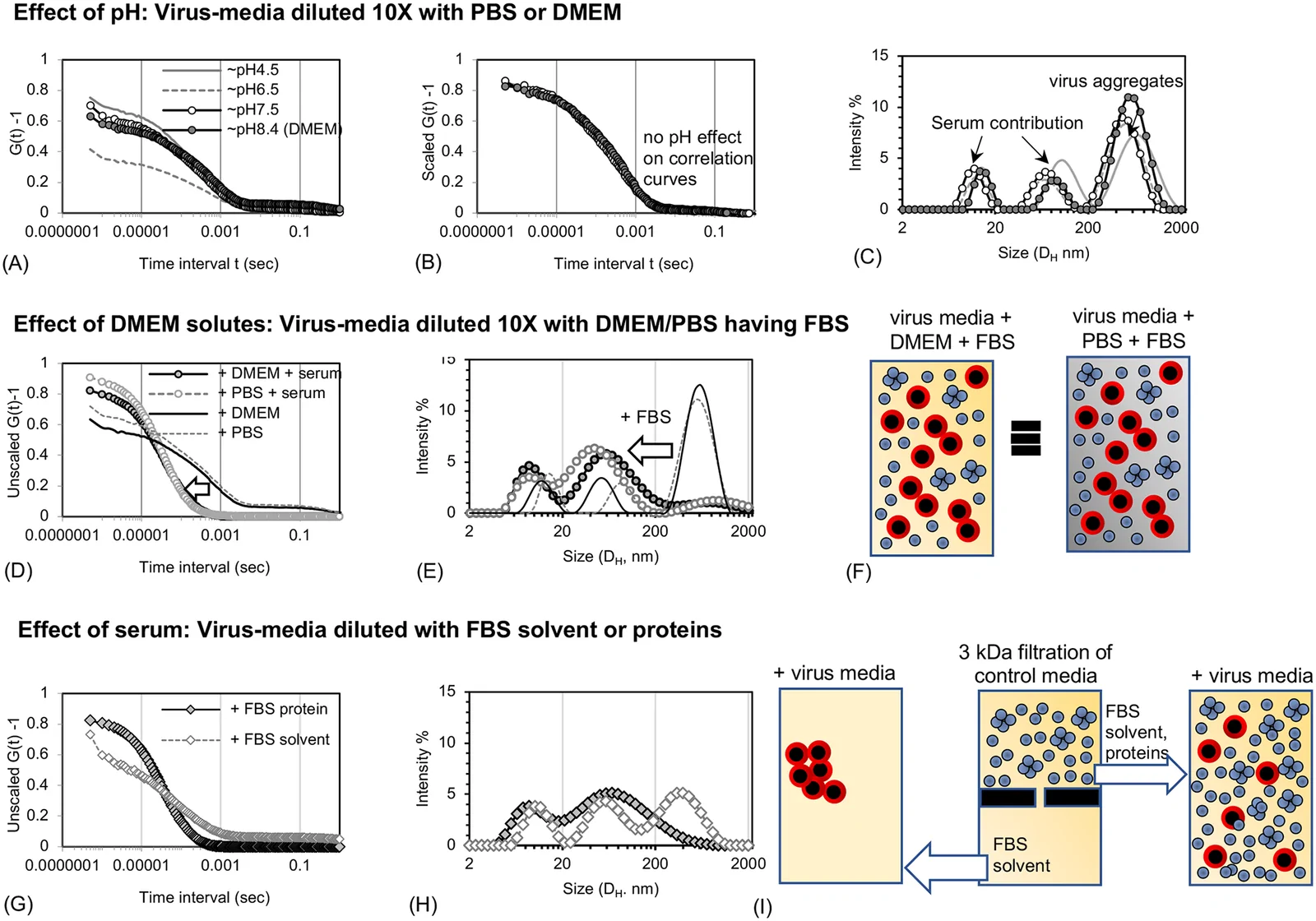
Component of culture media dispersing aggregated virus. [A] DLS curves of virus media diluted with PBS to pH 4–7.4 and with DMEM to pH 8.5. [B] Scaled DLS curves at different pH with the overlap indicating similar distribution of diffusion sizes. [C] Scaled D_H_ histograms showing comparable levels of aggregated virus at the different pH tested. [D] DLS curves comparing virus-media before and after dilution with either DMEM or PBS, without and with FBS supplementation. [E] DLS histograms showing similar diffusion peaks for virus media diluted with PBS or DMEM, but the aggregate peak dispersing when either solvent included 10% FBS. [F] Schematic highlighting that FBS was influencing virus dispersal, irrespective of DMEM or PBS being the solvent. [G] DLS curves showing that control media containing only FBS solvent (no proteins) did not shift the virus media curves (i.e., did not change virus aggregation), whereas control media also containing FBS proteins left-shifted the DLS curves. [H] Corresponding D_H_ histograms showing that the FBS proteins and not the FBS solutes were dispersing the ~ 500 nm virus aggregates. [I] Schematic summarizing that control-media was filter-centrifuged (3 kDa cut-off) to obtain filtrate containing FBS solvent and a concentrate retaining the FBS proteins, wherein the latter dispersed virus aggregates but the former did not.

**pH**: The VSV-G envelope protein is known to exhibit a pH-dependent transition from a pre-fusion state above pH 7 to a membrane-binding state that increases with acidic pH and a post-fusion state by pH 5.5 [25–28]. We checked if the aggregation and dispersal of the virus was a reflection of pH-induced changes in the virus envelope protein. However, virus aggregates were observed in virus media diluted in PBS and pH adjusted from 4.5–7.4 (Fig 3A–C). Aggregates were also observed in virus media diluted with DMEM at ambient CO_2_ levels to achieve a pH of ~8.5. The dispersion of the viral aggregates did not appear triggered by pH induced changes to the virus envelope proteins.

**Dilution**: Poorly solvated proteins and particles tend to show a large diffusion size in DLS at low concentrations. The DLS data of virus media showed a consistent presence of aggregated species at multiple dilutions (S2 Fig), confirming it was not a dilution artifact. Media containing bald virus, on the other hand, displayed a different dilution behavior (S2 Fig).

**DMEM and FBS solutes**: Virus media diluted in either PBS or DMEM had similar correlation curves (Fig 3D) with pronounced ~500 nm D_H_ peaks (Fig 3E), indicating that the solute components of DMEM were not involved in the aggregate dispersal. The DMEM solutes include 1.8 mM Ca, glucose, and phenol red.

**FBS solutes and proteins**: Virus media diluted with either PBS or DMEM containing 10% vol/vol FBS, showed dispersal of the virus aggregates with signature leftward shifting of the DLS curves (Fig 3D) and loss of the ~ 500 nm D_H_ peak (Fig 3E). A component of the FBS solution was contributing to the dispersal of the virus aggregates, and we determined if it was coming from the solution proteins or solutes. FBS solution consists of serum proteins (~42 mg/mL) [29] in a solvent containing small solutes such as urea, cholesterol, bilirubin, lipids etc which are < 3 kDA. We determined if the FBS solvent or the FBS serum proteins were contributing to the virus dispersal. Control media (DMEM + 10% FBS) was filter-centrifuged with a 3 kDa filter membrane to separate FBS proteins from the FBS solvent that filtered through. Virus media diluted with the retentate FBS proteins resuspended in PBS showed a dispersal of the virus aggregates with leftward shift of DLS curves (Fig 3G) and loss of the aggregate peak (Fig 3H). The filtrate containing FBS solutes did not disperse the aggregate (Figs 3G, H). These observations suggesting the FBS proteins are dispersing the virus are schematized in Fig 3I.

**Relative amount or concentration of serum proteins**: Since virus media itself contains serum proteins but permitted virus aggregation, we determined if there was a preferred serum concentration or preferred serum/virus ratio for virus aggregates to disperse. Different % volumes of FBS were added to different dilutions of virus media in PBS (Table 1). The ratio of virus in the aggregated versus free state was monitored by I_agg_/I_free_, the ratio of the intensity of the aggregate peak to that of the free virus/serum-aggregate peak in DLS histograms, and which roughly captures the total volume of the aggregate species to the total volume of free virus (See S3 Text). The fraction of virus contained in the aggregate is a more important parameter than the number of aggregates since the aggregates are not irreversibly sequestered entities but can be dispersed into free virus, and because the aggregates themselves contribute to infection as shown later. We note that the %vol of serum reported in Table 1 includes both the FBS solution already present in virus-media and that supplemented after.

**Table 1:** Level of virus aggregation observed at different virus dilutions and % volume of serum. The extent of virus aggregation was quantified by the ratio of intensities of the ~ 500 nm virus-aggregate and of the ~ 60 nm serum/single-virus peaks in the DLS histogram (I_agg_/I_free_). The regions with high serum and low virus amounts (lower right corner of table) had lower ratio of virus aggregates, irrespective of concentrations of serum or virus (grey cells). The cells in yellow are different dilutions of virus media with no additional serum supplementation. The combinations of serum and virus in dark grey cells had little to no aggregation.

Virus dilution	Serum vol/vol			
	1%	2%	5%	10%
1X				0.964 ± 0.476
2X			1.166 ± 0.105	0.766 ± 0.055
5X		0.979 ± 0.112	1.088 ± 0.042	0.465 ± 0.096
10X	1.983 ± 0.317	0.609 ± 0.021	0.350 ± 0.069	0.145 ± 0.061
20X	0.752 ± 0.107	0.568 ± 0.044	0.140 ± 0.058	0.130 ± 0.032

The %vol of serum required to disperse virus aggregates decreased with the amount of virus (i.e., increased dilution of virus media in Table 1) I_agg_/I_free_, suggesting that it is the serum/virus ratio and not the serum concentration responsible for aggregate dispersion. The reliance on serum:virus ratios indicate a likely competition binding between serum-virus and virus-virus associations that is dispersing the viral aggregates. To check on the robustness of the serum:virus dependence, 10X diluted virus media was filter-centrifuged with a 10 kDa membrane that retains serum proteins and virus (S4 Fig). The retentate having several-fold higher serum concentration but same serum:virus ratio still contained aggregated virus. Ultracentrifuged virus solution has minimal serum proteins in it and an I_agg_/I_free_ ~ 10, which reduces to <0.5 when the equivalent of 10% vol serum is added (S5 Fig).

### Ca^2+^ induced aggregation of VSV-G enveloped HIV-1 at pH > 7.4

**Fig 4:**
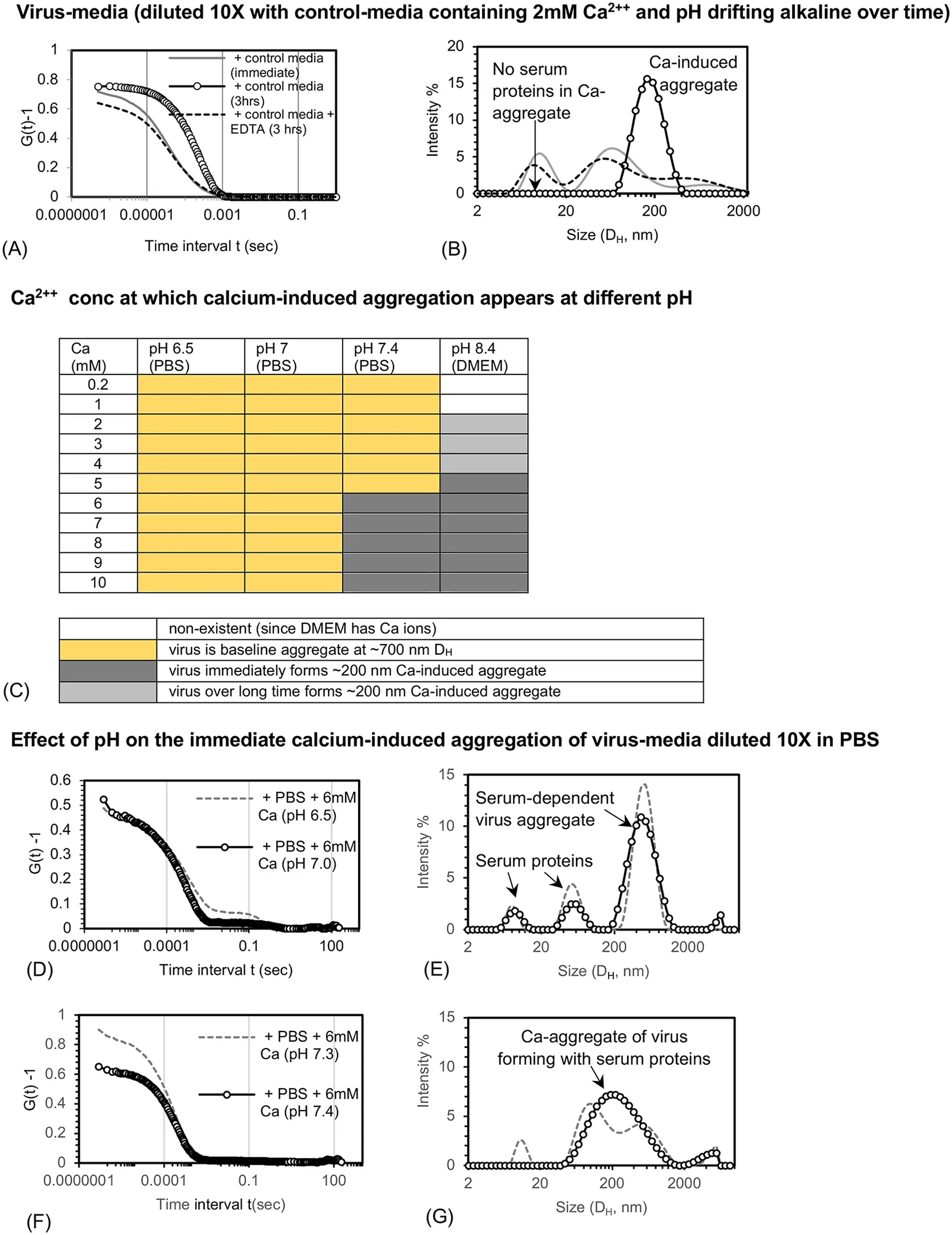
Ca^2+^-induced aggregation of VSV-G env virus occurs at pH > 7.4. [A] DLS curves of virus media diluted in control media take on a different form at around 3 hours, which is prevented when EDTA is present in solution. Control or culture media has ~ 2mM Ca ions which can be sequestered by EDTA, and pH of control media rises to > 7.4 at 3 hours. [B] Corresponding histogram showing that virus media diluted in control media has virus in dispersed state initially but forms a ~ 200 nm aggregate over 3 hours with loss of freely diffusing serum proteins. The addition of EDTA prevents transition to the ~ 200nm aggregate. [C] Effect of pH and Ca^2+^ concentration on the immediate- and long- term formation of Ca-aggregates. Virus media here is diluted in PBS, and therefore has a baseline ~500 nm aggregate in the absence of Ca-induced aggregation. [D, E] DLS correlation curves and histograms of the virus media in Fig. 4C at 6 mM Ca and pH < 7.3, showing no Ca-induced aggregation of virus and showing freely diffusing serum proteins. [F, G] DLS correlation curves and histograms of the virus media in Fig. C at 6 mM Ca and pH approaching 7.4 showing the transition to Ca-induced aggregation of virus with loss of free serum proteins.

We observed that when virus media was diluted 10X in control media and stored > 2 hours with pH drifting above 7.4, a different type of diffusion species appeared. It had a single D_H_ peak of ~200 nm, larger than free virus, and the separate diffusion peaks of free serum proteins disappeared, suggesting that proteins and virus were part of the ~ 200 nm species. (Fig 4A, B). The addition of EDTA prevented the formation of this species, indicating that Ca^2+^ ions were critical for its formation (Fig 4A, B). In addition, pH appeared to be critical for its formation (Fig 4C). 10X PBS-diluted virus media was set to different pH. As long as the pH was below 7.4, Ca^2+^ supplementation up to 10 mM did not induce formation of the ~ 200 nm species (Fig 4C). When pH was above 7.4, addition of 6 mM Ca^2+^ produced the ~ 200 nm species immediately. At pH 8.5, ~ 5 mM Ca^2+^ was sufficient to induce immediate virus aggregation. However, as long as the pH was above 7.4, 2 mM Ca^2+^ was sufficient to induce formation of the species over the course of two hours, (e.g., Fig 4A, B). Figs 4D-G shows DLS correlation and histogram curves of virus media at select pH from Fig 4C. The ~ 500 nm D_H_ aggregated species features prominently at acidic pH. The levels of ~500 nm aggregates and that of free proteins starts diminishing, however, around pH 7.3 ± 0.15. By pH 7.4, these are replaced by a single 200 nm D_H_ species. We refer to this diffusing conglomerate of virus and serum proteins that only forms at pH > 7.4 and with Ca^2+^ ions as the Ca-induced aggregate. Control media with serum proteins also displayed Ca^2+^-induced aggregation but at pH > 6.5 (S6 Fig).

### Three states of VSV-G enveloped HIV-1 respond differently to antibody, imaging, and infectivity studies

It appears that the VSV-G envelope lentivirus can exist in three diffusion states within the confines of cell culture conditions (summarized in Table 2). At low serum/virus conditions, and at Ca^2+^ < 2 mM or pH < 7.4, the virus exists in a self-aggregated with a co-existing free state. At high serum/virus conditions, but still with Ca^2+^ < 2 mM or pH < 7.4, the virus dispersed from the aggregated state. If the virus is dispersed by serum proteins, it is possible that the virus is not free but is coated by serum proteins. When Ca^2+^ > 2 mM and pH > 7.4, virus in media transitions to diffusing as a smaller aggregate formed with serum proteins, which we refer to as Ca-induced aggregation.

**Table 2:** A summary of the three states of the virus. The virus is dispersed at high serum/virus ratios and aggregated at low serum/virus ratios. Above pH 7.4, the virus state becomes sensitive to Ca ions. The listed calcium ions concentrations are those beyond which rapid (< 1hr) and slow (> 2.5 hrs) Ca-induced aggregation of virus and serum occurs.

Serum/virus	Time	Ca insensitive		Ca sensitive			
		pH 6.5	pH < 7.4	pH 7.4		pH 8.4 (DMEM)	
Low	<1hr			≤ 5 mM	≥ 6mM	≥ 2 mM	≥ 5mM
	>2.5hrs			≤ 5 mM	≥ 6mM	≥ 2 mM	
High	<1hr			≤ 6 mM	≥ 7mM	≥ 2 mM	≥ 3 mM
	>2.5hrs			≤ 5 mM	≥ 6mM	≥ 2 mM	
60 nm Dispersed virus							
~ 200 nm * Ca-induced aggregate							
> 400 nm Serum-dependent virus aggregate							

*The size can be larger than 200 nm if kept overnight or the pH is higher than 7.4

**Fig 5:**
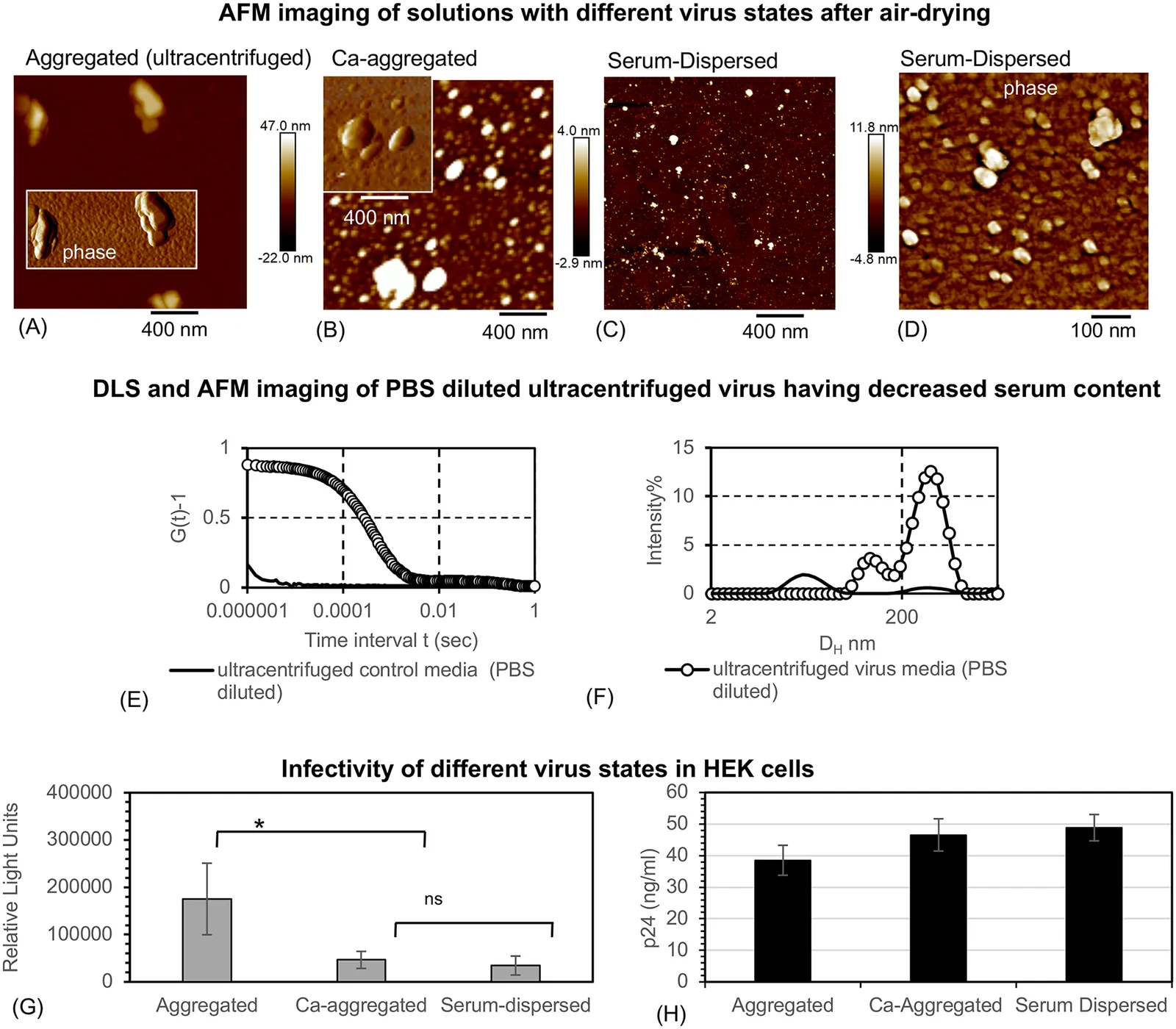
AFM imaging and Infectivity of the three virus states: [A] AFM dry imaging of ultracentrifuged virus media having negligible serum proteins which shows virus aggregates. The height map is shown alongside. [B] AFM dry imaging of virus media having Ca-induced aggregates which display 200 nm. [C] AFM dry imaging of virus media having high serum/virus content which show dispersed virus. [D] Zoomed iin AFM phase image of [C] where the imaged virus appears to have protein adsorbed on the virus surface (white box). [E] DLS correlation curves of resuspended pellets from ultracentrifuged virus media and control media which has insignificant signal from control media serum proteins. [F] DLS histograms corresponding to the correlation curves in [E]. [G] Infectivity assay of virus in different states determined in HEK cells by luciferase activity. [H] p24 assay of the virus solution when in different states.

The three states could be distinguished with AFM imaging (Fig 5A–D). For imaging the aggregate state that occurs in low serum/virus conditions, we imaged virus media subject to ultracentrifugation and resuspended in PBS. Ultracentrifugation removes most of the serum proteins, shown here by the loss of the serum diffusion peak in the purified virus media and by the loss of scattering in the control media subject to similar treatment (Fig 5E and 5F). Removal of serum proteins prevents rearrangement of the virus state on the AFM surface as the virus media is dried and serum proteins crowd on the drying virus. Both DLS correlation curves and histograms and AFM images of the ultracentrifuged virus media show virus aggregates in the ~ 400 nm range (Fig 5A, 5E and 5F). AFM imaging of virus media diluted in PBS pH 7.4 with Ca^2+^ ions had the ~ 200 nm aggregates similar to the size of Ca^2+^-induced aggregates observed in DLS (Fig 5B). At times a mix of virus and serum proteins could be distinguished in these aggregates (inset, Fig 5B). AFM imaging of virus media diluted in serum-supplemented PBS (no Ca^2+)^ produced dispersed particles in the ~ 100 nm size range (Fig 5C). Phase images showed the presence of serum proteins on the virus (Fig 5D). Histograms of the particle diameters from AFM images of the virus in the three states are summarized in S7Fig.

While small deviations from homeostatic conditions (pH > 7.4, Ca^2+^ > 2mM, serum levels within physiological range) produced large and defined changes in the diffusion states of the virus, we asked if these shifts were relevant to virus virulence. HEK cells were infected with virus in one of the three states. Luciferase activity resulting from integration of the viral gene was measured as indicator of infectivity. The aggregated state of the virus was found to have significantly higher infectivity than Ca- aggregated and serum-dispersed states (Fig 5G). The higher infectivity of the aggregate state confirms several of our findings including that the envelope proteins on the virus are exposed and available for binding. The lower infectivity of the dispersed state is consistent with the implications of our study that serum is coating the virus and likely preventing its uptake by cells. It is also possible that the implied agglomeration of serum, Ca, and virus in the Ca-induced state is lowering the availability of virus for infections. We note that ELISA assay picked a lower p24 count for virus media containing aggregated virus compared to the same media having supplemented calcium or serum to produce the Ca-induced aggregated and serum-dispersed states, respectively (Fig 5H). It is possible that the aggregated state releases p24 less effectively than the other states of the virus.

**Fig 6:**
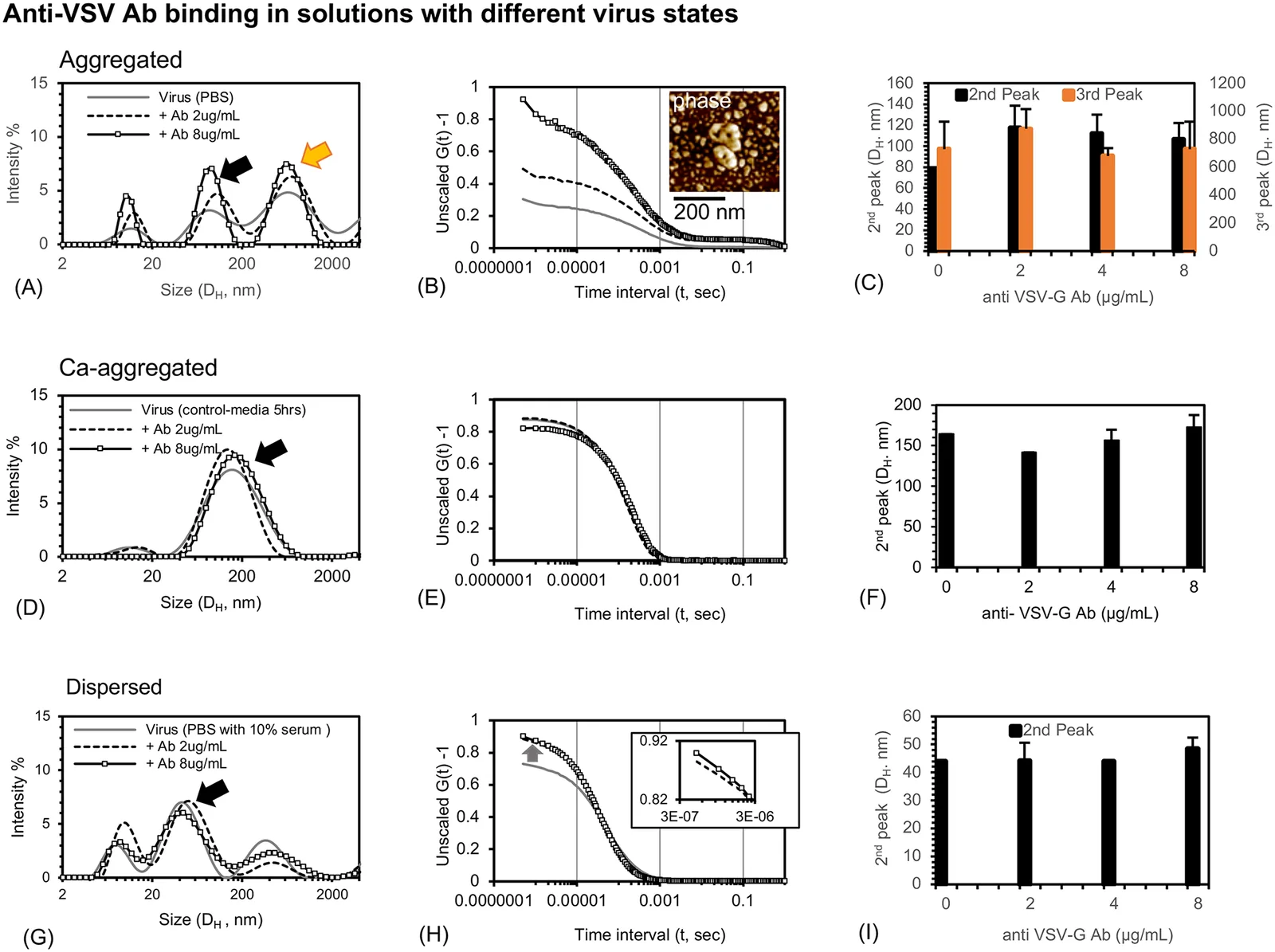
Changes in the DLS profile of virus in the three diffusion states following the addition of different amounts of antibodies (Ab): [A] Addition of anti- VSV-G antibodies to virus media diluted in PBS pH 6.5 and containing virus aggregates does not reorganize the diffusing species in DLS. [B] DLS correlation curves showing that addition of anti- VSV-G antibodies to virus media containing aggregates changes the scattered intensity but not the shape of the DLS correlation curve. Inset is an AFM phase image of the antibody-supplemented virus media showing antibodies covering virus particles. [C] Averages of hydrodynamic sizes of the second (serum aggregates) and third (virus aggregates) diffusion peaks following addition of different amounts of antibodies to virus media (n = 3). [D] Addition of anti- VSV-G antibodies to virus media containing Ca-induced aggregates shows no change in the diffusion peaks and appearance of a free antibody peak. [E] DLS correlation curves not affected by addition of antibodies to virus media containing Ca-induced aggregates. [F] Averages of the diffusing sizes obtained after antibody addition to Ca-induced virus aggregates. [G] Addition of anti- VSV-G antibodies to virus media containing dispersed virus does not change the diffusing species in DLS. [H] DLS correlation curves following addition of antibodies to virus media containing dispersed virus with an apparent increase in the contribution from the smaller diffusing species. [I] Averages of the hydrodynamic sizes obtained after antibody addition to dispersed state of virus in virus media, showing no systematic change in the sizes of the dispersed virus peak (arrow in Fig. 6G).

Finally, we checked the response of virus in the three states to antibody addition. For instance, would antibodies destabilize the solution states to different extents or produce a mass agglutination? Fig 6 shows representative histograms, correlation curves, and size averages obtained when increasing amounts of antibodies were added. For all three states, the addition of antibodies did not change the distribution of virus sizes (Fig 6A-I). In the case of aggregated virus, there was an increase in the net scattering intensity observed (Fig 6B) and AFM images showed virus surface speckled with antibodies (Fig 6A, inset), consistent with earlier observations that antibody could interact and pullout virus in the aggregated state. In the case of Ca-induced aggregates, the addition of antibodies did not disrupt the diffusing species or change its size (Fig 6D-F). No separate diffusion peaks appeared from free antibodies either, suggesting that the antibodies were likely integrating into the ~ 200 nm diffusion peak (Fig 6D). In the case of serum-dispersed virus, the addition of antibodies seemed to increase the level of free proteins in solution, more clearly seen with the correlation curves (Fig 6H). There was no change in the size of the dispersed virus peak, which is expected if the antibodies are remaining free in solution (Fig 6G,I).

### Glycosylation influences diffusion states of VSV-G env HIV-1 virus

**Fig 7:**
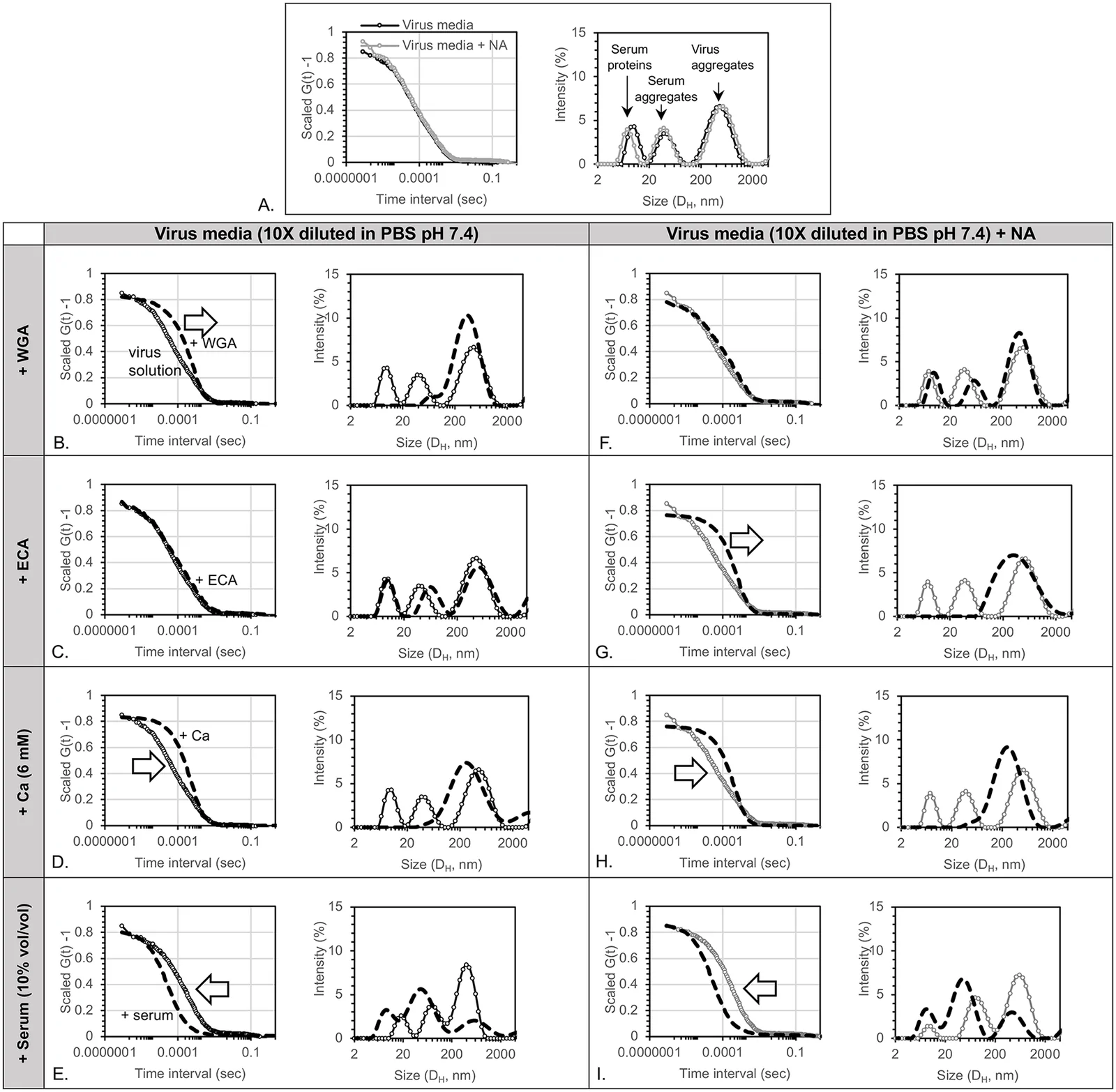
Comparing lectin and aggregation characteristics of virus media having aggregated virus before and after exposure to the SA-cleaving glycosidase Neuraminidase (NA). [A] DLS correlation curves (left) and corresponding D_H_ histogram (right) of virus media diluted 10X with PBS pH 7.4 and containing virus aggregates, for without and with NA exposure. There is no change in the correlograms and histograms following NA exposure. [B – E] DLS correlation curves (left) and corresponding D_H_ histogram (right) of virus media after exposure to: SA-lectin WGA which produces agglutination (B), the gal-lectin ECA which does induce agglutination (C), 6 mM Ca ions which induces Ca-dependent aggregation (D), and serum proteins which induce dispersion of virus aggregate (E). The dashed plots are DLS data of virus media after exposure to above agents. [F-I] DLS correlation curves (left) and corresponding D_H_ histogram (right) of virus media cleaved with NA after exposure to: WGA which shows no change (F), ECA which agglutinates the species (G), 6 mM Ca ions which induces aggregation (H), and serum proteins which induce aggregate dispersion (I). The dashed plots are DLS data of NA-cleaved virus media after exposure to above agents.

We investigated the extent to which the virus glycosylation determined its diffusion state. Neuraminidase (NA) was incubated with virus media diluted 10X in PBS to cleave the terminal SA residues of the complex N-glycans on the virus VSV-G envelope proteins and likely on serum proteins as well (Fig 7A). Removal of SA residues in complex N-Glycans exposes Gal residues (Fig 1A). SA cleavage from virus glycan was confirmed by loss of agglutination with SA lectin WGA (Fig 7B, F), and appearance of agglutination with galactose lectin ECA (Fig 7C, G). Agglutination of virus media by lectins was evident in DLS by the rightward shift of the DLS correlation curves and by the coalescing of the size histogram into a single strong peak. NA treatment did not change the aggregated diffusion state of the virus (Fig 7A). Neither did it diminish the switching to the Ca^2+^-induced aggregation state upon Ca^2+^ supplementation (Fig 7D, H) nor negate the dispersion of virus aggregates with serum addition (Fig 7E, I). It is possible that NA treatment only removed SA from the surface of the virus aggregates (virus still aggregated after NA treatment in Fig 7A), and this was not sufficient for influencing the solution state of the virus.

**Fig 8:**
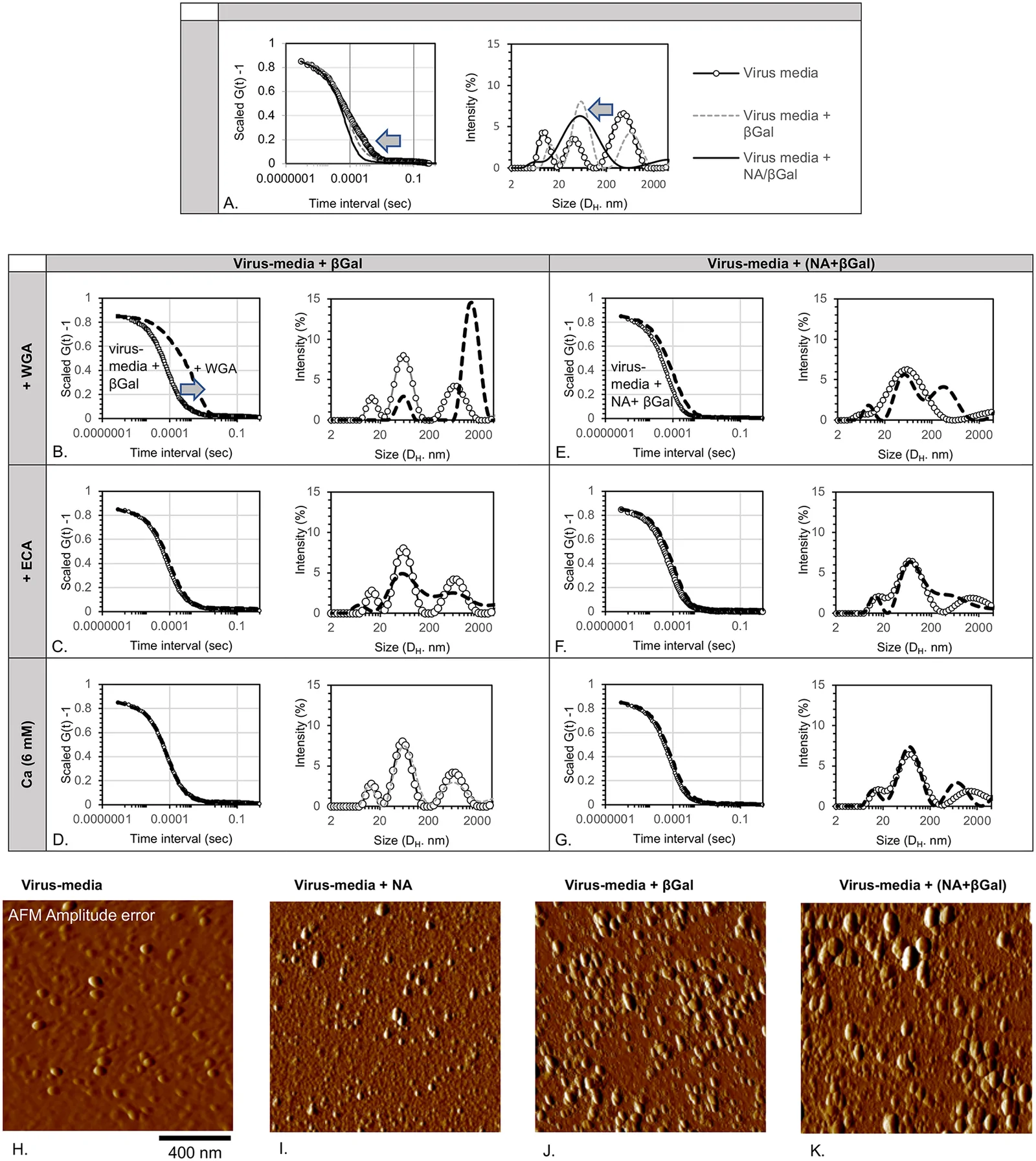
Comparing lectin and aggregation characteristics of virus media with aggregated state of virus before and after exposure to β-galactosidase (β-gal) (left panels) and to a mix of β-gal and Neuraminidase (NA) (right panels). [A] DLS correlation curves (left) and D_H_ histogram (right) of virus media diluted 10X with PBS pH 7.4 before and after treatment with β-gal and (β-gal + NA). There is an increasing loss of the late relaxation times and large diffusion peaks. [B – D] DLS correlation curves (left) and corresponding D_H_ histogram (right) of β-gal cleaved virus media after exposure to: SA-lectin WGA (B), gal-lectin ECA (C), and 6 mM Ca^2+^ ions (D). The dashed plots are DLS data of virus media after exposure to above agents. [E-G] DLS correlation curves (left) and corresponding D_H_ histogram (right) of (β-gal + NA) cleaved virus media after exposure to: WGA (E), ECA (F), and 6 mM Ca^2+^ ions (G). [H – K] AFM amplitude error images comparing the surface adsorption of virus media before (H) and after exposure to NA (I), β-gal (J), NA + β-gal (K).

Virus media diluted 10X in PBS was incubated with β-galactosidase (β-gal) and with a mixture of NA + β-gal (Fig 8). There was partial and complete loss of the aggregated state, respectively, evident from the left-shifting of the DLS correlation curve (Fig 8A). Virus media exposed to β-gal agglutinated strongly with WGA (SA and GlcNAc lectin) and not with ECA (Galactose lectin), consistent with the removal of galactose and exposure of GlcNAc residues and from the presence of SA residues (Fig 8B, C). Virus media exposed to NA + β-gal agglutinated to a lesser extent with WGA and did not agglutinate with ECA, consistent with the removal of both SA and Gal residues and exposure of GlcNAc residues (Fig 8E, F). Glycosidase-treated virus media in both cases did not transition to the Ca-induced aggregated state (Figs 8D, G). Since the glycosidase-treated media already had virus in the dispersed state, there was no need to further test serum dispersion. β-gal and NA + β-gal treatments were both effective in removing the aggregate states of the virus, but it is not clear if the appearance of GlcNAc residues or the effective removal of SA and Gal residues was responsible for these effects.

AFM imaging confirmed that β-gal and NA + β-gal treatment changed the surface of the viruses similarly, but different from untreated and NA-treated virus (Fig 8H–K). Virus in untreated and NA-treated media did not adsorb strongly to mica surface. Viruses cleaved by β-gal and NA + β-gal, however, adsorbed densely on the mica surface forming surface clusters, indicating that the surface biophysics of these viruses have changed to similar extents, possibly by the exposure of the GlcNAc residues or by the improved removal of SA residues.

## Discussion

Our goal was to determine how N-glycosylation, particularly of the complex N-glycans type, influenced the state of biological units like viruses in different solution conditions. HIV-1 lentivirus having VSV-G envelope proteins from the Vesicular Stomatitis Virus (VSV) display complex type N-glycans. We report that the virus can exist in solution in one of three diffusion states: self-aggregated, aggregated with serum and by Ca ions, dispersed by and likely coated with serum. The environmental triggers for shifting from one state to another occurs at physiological conditions. Changes in diffusion states are accompanied by several fold changes in virus infectivity. The solution states and transitions between them are lost when the terminal SA and Gal residues from the virus complex N glycans are cleaved.

HIV-1 lentivirus have been garnering attention in gene and cell therapy because DNA delivered via lentivirus infection can integrate into the genome of cells and the cells do not have to divide for the DNA to enter the nucleus [30]. However the selectivity of the HIV-1 gp160 env limits the range of cells that can be infected, and the lentivirus is pseudo-typed with envelope proteins from viruses with broad tropism to overcome this limitation [31]. The VSV virus has a broad tropism, infecting both insect and animal hosts [32,33]. VSV-G, the glycosylated env of VSV, binds the low-density lipoprotein receptor (LDLR) ubiquitous in animal and insect cells [34,35]. HIV lentivirus with VSV-G env has been used to generate stable genetically modified cell lines for research [36–39]. Replication-deficient and self-inactivating lentivirus with VSV-G env are also being researched as gene delivery vehicles in biomedical applications such as viral oncolysis, vaccination, gene therapy, and CAR T cell therapy [12,40–43]. VSV-G has also been used to pseudotype adenoviral and retroviral vectors [39,44]. Given the wide use of lentivirus with VSV-G env, and of the VSV-G env itself, understanding how the solution conditions affect the virus state – its aggregation, diffusion size, surface properties, and infectivity – is critical for planning the efficiency and outcomes of the gene transport and delivery in these applications.

**Fig 9:**
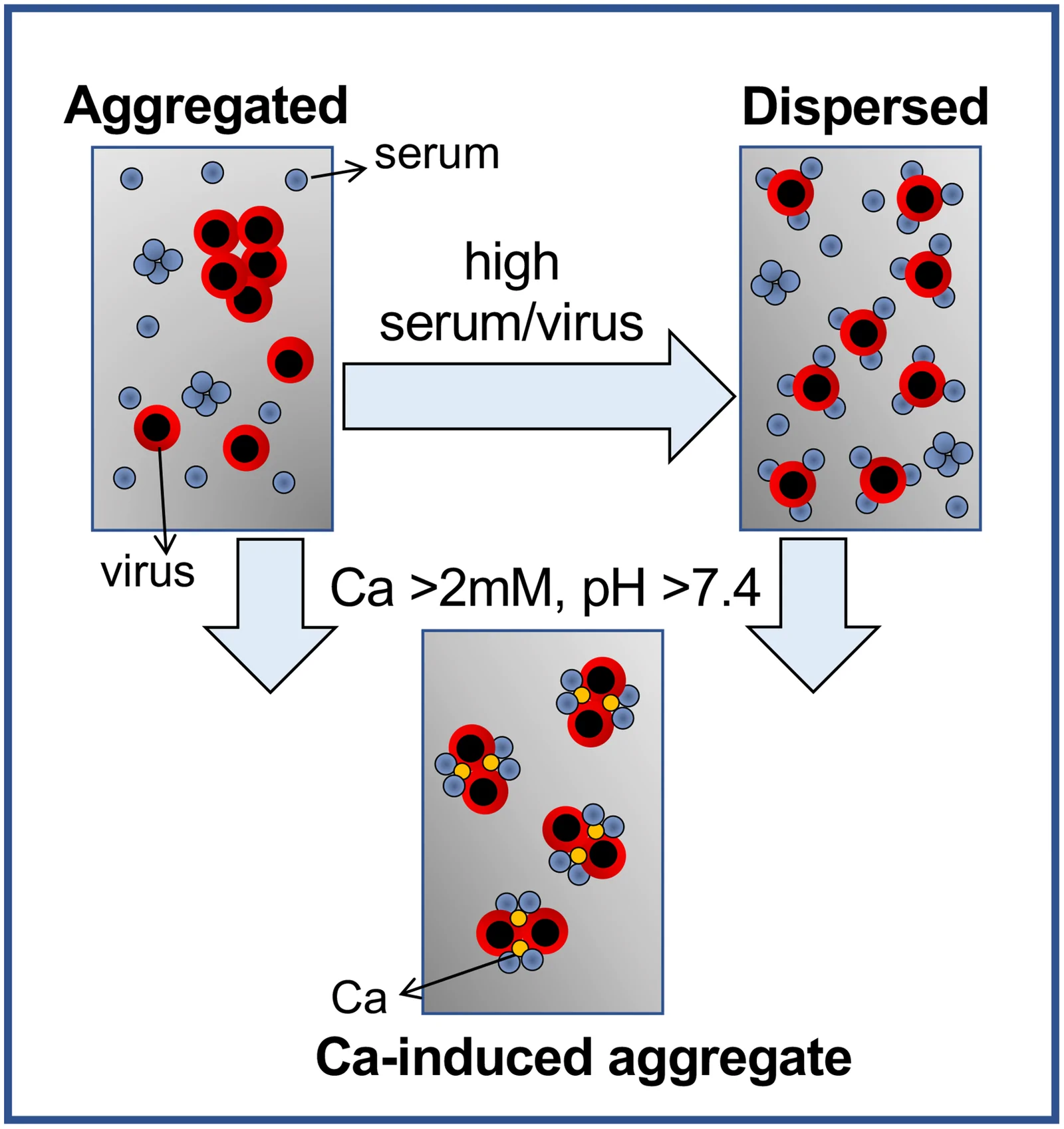
Schematic summarizing three solution states of VSV-G env HIV-1 lentivirus inferred from the study findings.

The following conditions and characteristics were observed for the three diffusion or solution states of the VSV-G env lentivirus which are summarized in a schematic in Fig 9. A significant fraction of the virus existed self-aggregated in solution as long as the serum/virus amounts were low, and as long as either the Ca^2+^ ion concentration was less than 2mM or the pH was less than 7.4. The self-aggregates were in the range of ~500 nm in AFM and DLS studies, appear to co-exist with free virus, can be bound and pulled out of solution by antibodies, and can be removed by filtration through 200 nm pore size with attendant loss of about half the p24 count. The aggregates appear to release p24 less effectively in ELISA assays, but are highly infective. In solution conditions where the serum/virus ratio increased but either the Ca^2+^ ion concentration remained less than 2mM or the pH remained less than 7.4, the aggregates get dispersed by the serum proteins. While we have not identified the type of serum dispersing the virus, the dependence of serum/virus ratio suggests that serum is competing for binding the virus interacting with other virus in the self-aggregates. The binding of serum to virus is also evident from AFM images where serum proteins are seen on the virus surface and from the loss of a separated free serum diffusion peak in DLS. Antibodies do not effectively agglutinate this state and the infectivity of virus in this state is several folds lower than in the aggregated state. The latter can be explained by the possible neutralizing effect of serum interacting with the virus. A third solution state of the virus occurs when both the Ca ions are above 2mM and the pH is above 7.4. The state produces a ~ 200 nm species in DLS and in AFM, likely comprised of serum, virus, and Ca ions, which we refer to as Ca-induced aggregates. The sequestration of Ca ions with EDTA prevents that formation of this state. The infectivity of these Ca-induced aggregates is again lower than that of the self-aggregated form and is on the order of the serum-dispersed state. Treatment of virus media having aggregated virus with Neuraminidase led to loss of terminal SA residues on the surface of the virus aggregates in media, but without altering the diffusion state of the virus and the ability to switch to others. However, removal of terminal galactose or of terminal SA and galactose, produced the loss of the aggregated state and of the ability to switch between states. The glycosylation of the virus appears relevant for the solution states it adopts in different solution conditions.

The conditions at which the VSV-G envelope HIV-1 lentivirus changes diffusion states do not appear to correlate with the conditions where VSV-G protein undergoes conformational changes. VSV-G envelope proteins in solution shift to a membrane-binding conformation as the pH decreasing below 7 [25–28]; The membrane-binding conformation allows the virus to penetrate intracellular endosomes at pH 4.5. It is not clear if theVSV envelope protein switching  configuration at acidic pH has any bearing on the virus switching diffusion states at pH 7.4 in the presence of Ca ions. Finkelshtein et al. [34] noted that the interaction of VSV-G lentivirus with soluble LDL receptors required the presence of Ca ions. It does not appear that this Ca requirement is related to the occurence of Ca-induced virus aggregation. Their study used purified virus and 1mM Ca ions, that latter being lower than the amount we found needed to induce the Ca-aggregated state. Finally, if the solution states of the VSV-G env lentivirus were determined by the complex N-glycans on the VSV-G env, then we would expect semblances of such solution behavior to also occur withVesicular Stomatitis Virus (VSV). Researchers have studied the aggregation of VSV virus and its impact on the evolution of virus infectivity and resistance [45–49]. Cuevas et al [47] reported VSV-G env proteins to be mediating the self-aggregation of VSV virus, which is consistent with our findings. However, Cuevas et al concluded that the VSV-G env was interacting with the phosphatidyl serine (PS) in the membrane lipids of neighboring virus. Cuevas et al also reported that VSV is more aggregated when purified than when in culture media, which is consistent with our findings that serum disperses the aggregated state. Our observation that serum is neutralizing the VSV-G env lentivirus also has parallels in observations that VSV virus is neutralized by human serum [50,51].

The solution behavior of a lentivirus displaying complex N-glycans on its env is very different than the solution behavior of the same lentivirus displaying mannose N-glycans on its env [52]. Earlier we reported that mannose-terminal lentivirus exhibit short-range, brittle, or Velcro-like self-adhesions in force spectroscopy, whereas SA-terminal lentivirus exhibit long-range, tough, or Slime-like adhesions in force spectroscopy, while GlcNAc terminal virus do not exhibit self-adhesion to each other [53–55]. Mannose-terminal and SA-terminal lentivirus were found self-aggregated in culture media, but the physical properties of their aggregation and the conditions that foster it were found different. Mannose-terminal virus aggregates can be sheared apart by filtration and pass through 0.2µm filters, whereas SA-terminal virus coagulate on a membrane filter. Unlike the SA-terminal VSV-G lentivirus, the mannose-terminal Gp160 env lentivirus was not dispersed by serum or was aggregated into a different form by Ca ions [52]. However, both the self-aggregated forms of the mannose- and of the SA- terminal virus were more infective than the dispersed forms. Consistent with the absence of self-adhesion observed in the force spectroscopy between two viruses with terminal GlcNAc residues [53], the exposure of GlcNAc residues in the VSV-G env and in the Gp160 env lentivirus [52] prevented their respective self-aggregation in solution.

The findings in our study have three broad implications. Firstly, if the terminal sugars on the virus are determining the diffusion state of the virus, then it is possible that other virus with terminal complex N-glycans could display such solution behavior. Secondly, if the triggers for switching solution state in VSV-G env HIV occur at physiological levels of pH, Ca ions, and serum, and if changes in the solution state drastically alter infectivity outcomes, then it is possible that cell culture and *ex vivo* and *in vivo* infection results may be altered by small drifts away from homeostatic conditions. Finally, since a virus in its transmission path travels through a broader range of pH, serum levels, and ion concentrations, it is possible that it switches solution states over the course of its transmission pathway, and this might confer advantages or vulnerabilities as it traverses that section of the transmission pathway.

## Supporting information

S1 Fig DLS of media from control transfections. (PDF)

S2 Fig DLS profiles of virus media and that from control transfection at different dilutions. (PDF)

S3 Text Inferred relation between the relative intensity of aggregated and free virus and the relative number of virus trapped in aggregated and free state. (PDF)

S4 Fig DLS profile of the concentrate and filtrate obtained when virus-media (diluted 10X in PBS) is filter-centrifuged through a 10 kDa membrane. (PDF)

S5 Fig DLS profile of purified virus after addition of FBS. (PDF)

S6 Fig DLS profile of control media supplemented with Calcium ions at different pH. (PDF)

S7 Fig Histogram of particle diameters observed in AFM imaging of the three states of the virus. (PDF)

S8 Fig DLS profile of virus media following PNGase treatment. (PDF)
