# Supplementary material for: HIV-1 lentivirus with VSV-G envelope: Complex N glycosylation governs shifts in aggregation and infectivity state at close to physiological pH, serum, and Ca2+ levels
Source: PLoS One. 2026 Sep 9;21(9):e0357567. doi: 10.1371/journal.pone.0357567 (PMC13557354; doi:10.1371/journal.pone.0357567)
Supplement: Supplementary file 1 [file pone.0357567.s001.pdf]

S1 Fig. DLS of media from control transfections

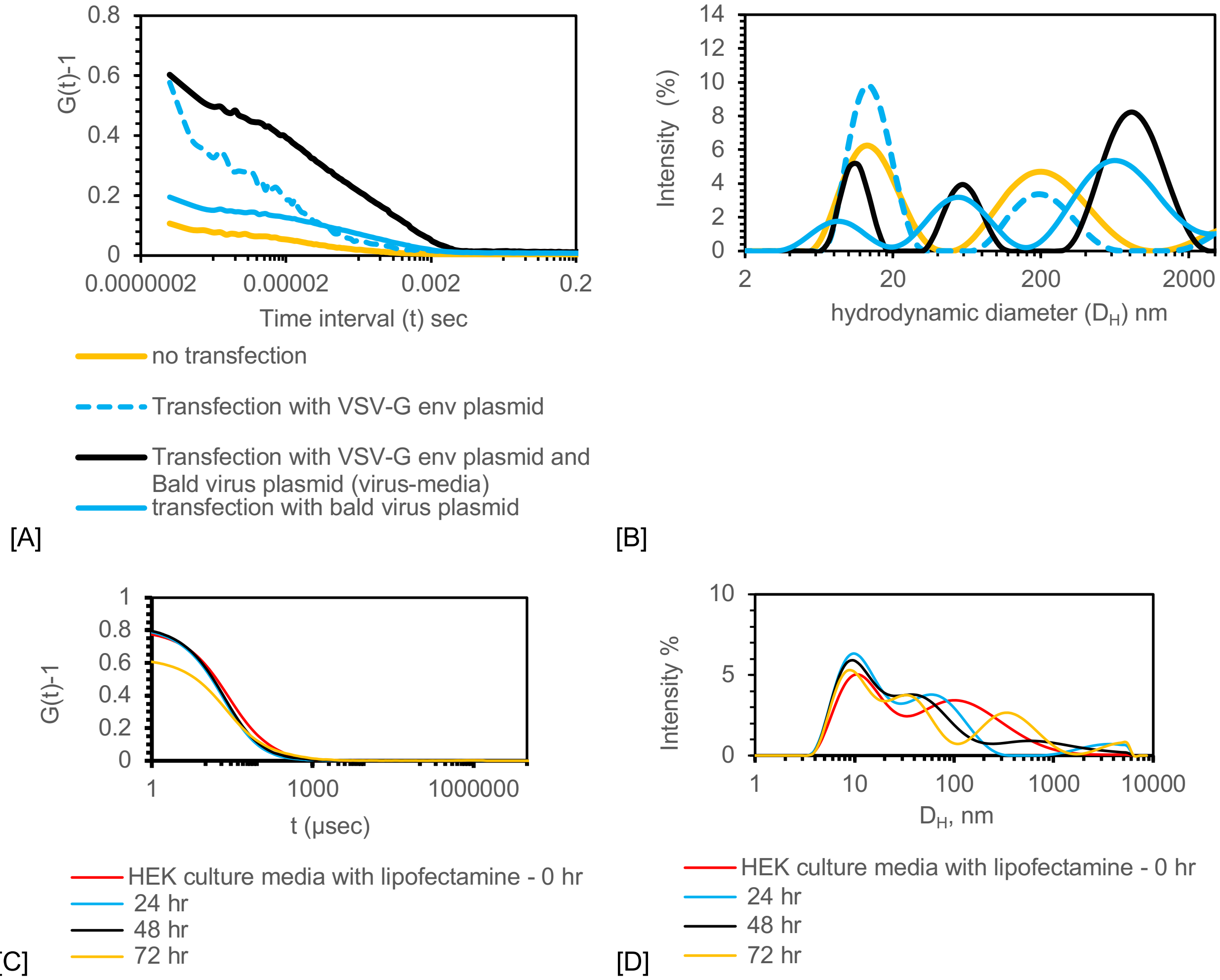

[A and B] To produce the virus, cells are transfected with plasmids for the envelop proteins (produces VSV-G proteins only) and for the provirus (produces bald virus with no envelop proteins). The DLS correlation curves and histograms in this figure compare the clarified media from HEK cells that were not transfected (yellow), transfected with both plasmids to produce the virus (black), transfected with the VSV-G envelop protein plasmid alone (blue dotted) and with the bald virus plasmid alone (blue). In all cases, cell culture media was clarified by centrifugation, diluted 10X in PBS, and examined with DLS. The correlation curves in [A] show a strong signal from a larger diffusing species only for the case of the virus media (i.e., provirus + VSV-G env plasmid transfection, red curve). The transfection with the Env plasmids showed a strong signal increase at lower diffusion sizes in the correlation curves, which were in the size range of free proteins in [B]. In other words, there was a likely increase in the VSV-G production. When transfected by the bald virus plasmid alone, the signal was weak, polydisperse, and only modestly higher than the no transfection control. [C and D] HEK culture media with lipofectamine transfection agent added to it measured at different times during cell culture.
