# Supplementary material for: HIV-1 lentivirus with VSV-G envelope: Complex N glycosylation governs shifts in aggregation and infectivity state at close to physiological pH, serum, and Ca2+ levels
Source: PLoS One. 2026 Sep 9;21(9):e0357567. doi: 10.1371/journal.pone.0357567 (PMC13557354; doi:10.1371/journal.pone.0357567)
Supplement: Supplementary file 2 [file pone.0357567.s002.pdf]

S2 Fig. DLS profiles of virus media and that from control transfection at different dilutions

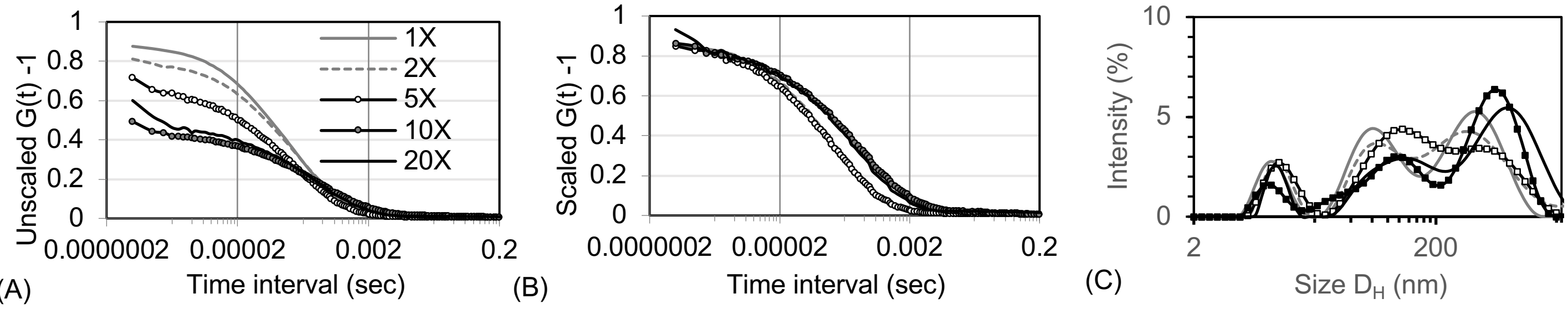

Tracking effect of dilution on virus aggregation with DLS: (A) Unscaled DLS curves from increasing dilution of virus media in PBS; (B) Scaled DLS curves from (A) showing the DLS curves are consistent with dilution but shift to the right for dilutions greater than 5X. (C) Histogram of D_H populations in solution showing consistent presence of an aggregated virus species (>500nm) at all dilutions, which have larger D_H at dilutions greater than 5X.

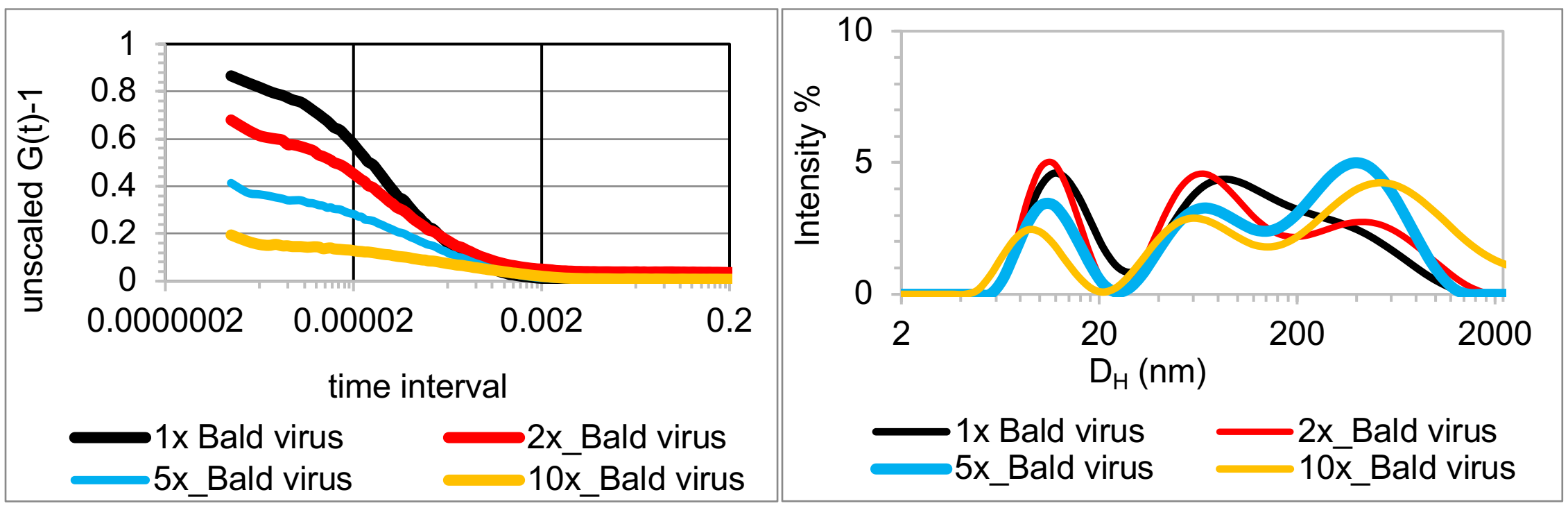

Dilution data of culture media with bald virus is different than the dilution data of virus media shown above. The relative contribution of the aggregate species is smaller at the lower dilutions and becomes unreliable at higher dilutions due to the lower signal.
