# Supplementary material for: HIV-1 lentivirus with VSV-G envelope: Complex N glycosylation governs shifts in aggregation and infectivity state at close to physiological pH, serum, and Ca2+ levels
Source: PLoS One. 2026 Sep 9;21(9):e0357567. doi: 10.1371/journal.pone.0357567 (PMC13557354; doi:10.1371/journal.pone.0357567)
Supplement: Supplementary file 3 [file pone.0357567.s003.pdf]

S3 Text: Inferred relation between the relative intensity of aggregated and free virus and the relative number of virus trapped in aggregated and free state

It can be argued that ratio of the intensities of the aggregate and monodisperse virus is a good indicator of the fraction of virus trapped in the aggregates.

Virus media shows three peaks: the first is ~10 nm that is attributable to free proteins; the second is ~ 80 – 100 nm attributable to aggregated proteins and free virus in media; and the third is ~ 300 – 800 nm which is being attributed to viral aggregates. The intensity contribution (I_i) of these peaks varies on the order of:

$$I_i \sim n_i \bar{I}_i \quad \text{-(1)}$$

Where n_i is the number of particles in that hydrodynamic size peak and the $\bar{I}_i$ is the intensity contribution per particle. When the particle is < 1/10th wavelength of the incoming light, Rayleigh scattering occurs, and here

$$\bar{I}_i \sim (d_i)^6 \quad \text{-(2)}$$

Where d_i is the particle diameter. Since the wavelength of light in the Zetasizer is 633 nm, such dependence is more applicable for the free protein diffusion peak and for small serum aggregates.

Viruses and aggregates have diffusion size on the order of 100 nm and for particle sizes > 1/10th wavelength of incoming light, scattering occurs in the Mie regime. Here the scattered intensity has a non-monotonic and lower power dependence on particle diameter. As per the instrument specifications (white paper on particle sizing from company website) on the values $\bar{I}_i$ for the sizes in the range of the aggregate peak (400 – 800nm) and the free virus peak (~100nm) are:

$$\frac{\overline{I_{agg}}}{\overline{I_{free}}} \sim 100 \quad \text{-(3)}$$

Where $\overline{I_{agg}}$ is the scattering intensity per particle having the diffusion size of the virus aggregate and $\overline{I_{free}}$ is the scattering intensity per particle having the diffusion size of the free virus. The number of aggregates and free viruses in solution can be obtained from the intensity histogram of particles sizes as

$$\frac{I_{agg}}{I_{free}} = \frac{n_{agg}}{n_{free}} * \frac{\overline{I_{agg}}}{\overline{I_{free}}} \sim 100 \frac{n_{agg}}{n_{free}} \quad \text{-(4)}$$

Where I_{agg} and I_{free} are the intensity of the free and aggregated virus peak in the DLS histogram and n_{agg} and n_{free} are the number of free and aggregated particles in solution.

However, since the virus particles in the aggregates remain dispersible, what is more relevant is the fraction of virus particles in the aggregates, because these can be released when the aggregate is dispersed. Let n_{v-agg} and n_{v-free} be the number of virus particles in aggregated and free states, and using a conservative estimate of 0.74 for packing density of sphere of the size of free virus into larger spheres of the size of virus aggregates [1, 2], we get:

$$\frac{n_{v-agg}}{n_{v-free}} \sim \frac{n_{agg}}{n_{free}} * 0.74 \left(\frac{D_{agg}}{D_{free}}\right)^3 \quad \text{-(5)}$$

Here D_{agg} and D_{free} are the hydrodynamic diameter of the aggregate and free virus particles. Using a commonly observed values of 500 nm and 100 nm, respectively for the aggregate and free virus particle hydrodynamic diameters, and substituting for $\frac{n_{agg}}{n_{free}}$ from Eq. 3, we get

$$\frac{n_{v-agg}}{n_{v-free}} \sim \frac{I_{agg}}{I_{free}} * .92 \quad \text{-(6)}$$

In other words, $\frac{I_{agg}}{I_{free}}$ for the dimensions of the virus and aggregate is a rough indicator of the ratio of virus particles trapped in the aggregated and dispersed state. The contribution of free virus particles can be overestimated in these estimations, since I_{free} has contributions from serum protein aggregates as well.

[1] A. Irannezhad, A. Baragry, D. Weaire, A. Mughal, S. Hutzler, Packing soft spheres: experimental demonstrations with hydrogels, European Journal of Physics 44(6) (2023) 065501.
[2] J.D. Bernal, A geometrical approach to the structure of liquids, Nature 183(4655) (1959) 141-147.
