# Supplementary material for: HIV-1 lentivirus with VSV-G envelope: Complex N glycosylation governs shifts in aggregation and infectivity state at close to physiological pH, serum, and Ca2+ levels
Source: PLoS One. 2026 Sep 9;21(9):e0357567. doi: 10.1371/journal.pone.0357567 (PMC13557354; doi:10.1371/journal.pone.0357567)
Supplement: Supplementary file 4 [file pone.0357567.s004.pdf]

S4 Fig. DLS profile of the concentrate and filtrate obtained when virus-media (diluted 10X in PBS) is filter-centrifuged through a 10 kDa membrane

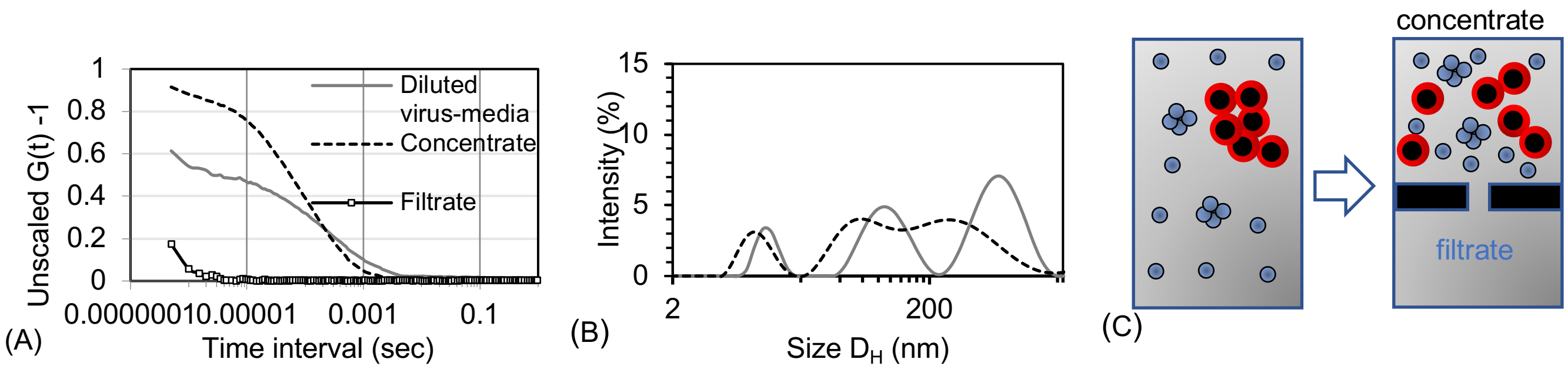

(A) Unscaled correlation curves of the diluted virus media, filtrate, and concentrate showing that not many particles came through the filtrate and that the intensity scatter from the concentrate increased due to the denser presence of particles there. (B) Histogram of the D_H populations in the virus-media and the 10 kDa concentrate showing the presence of aggregated virus in both. The concentrated virus solution in this experiment is similar to the lower dilutions of virus media in Suppl. Fig. 3C, attesting to the robustness of the DLS histograms. (C) Schematic of the centrifugation-filtration experiment and the results.
