# Supplementary material for: HIV-1 lentivirus with VSV-G envelope: Complex N glycosylation governs shifts in aggregation and infectivity state at close to physiological pH, serum, and Ca2+ levels
Source: PLoS One. 2026 Sep 9;21(9):e0357567. doi: 10.1371/journal.pone.0357567 (PMC13557354; doi:10.1371/journal.pone.0357567)
Supplement: Supplementary file 5 [file pone.0357567.s005.pdf]

S5 Fig. DLS profile of purified virus after addition of FBS

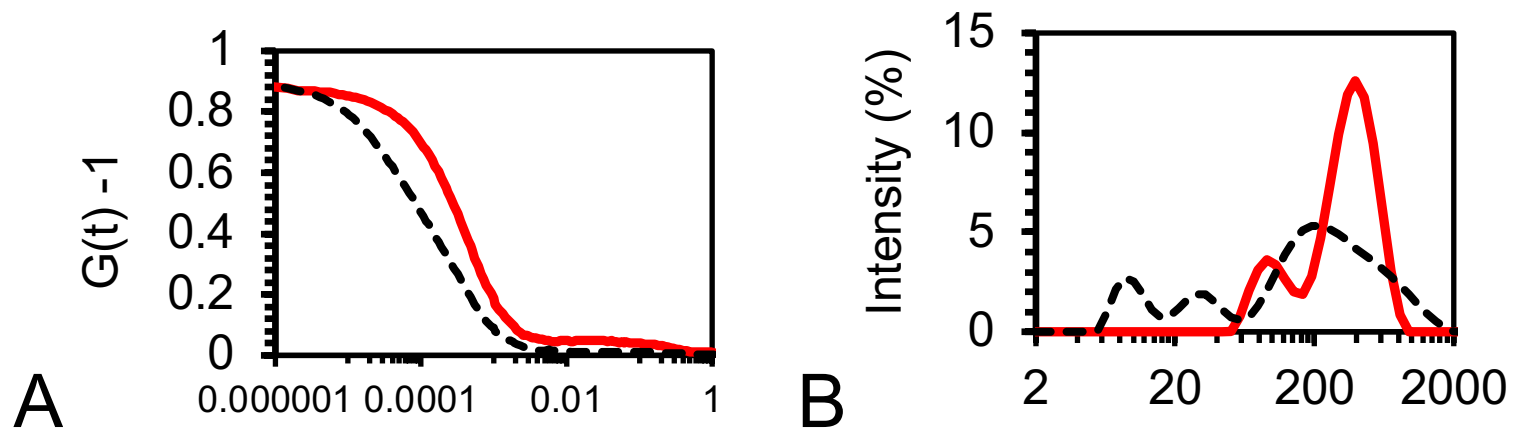

Ultracentrifuged virus solution (red curves in histogram and correlation curves) has minimal serum proteins and shows nearly a single 400 nm virus species, which is at a size larger than a free virus. The addition of serum proteins (3.78 mg/ml) produces a diffusion peak in the 100 nm range, consistent with the dispersion of virus aggregates with serum addition
