# Supplementary material for: HIV-1 lentivirus with VSV-G envelope: Complex N glycosylation governs shifts in aggregation and infectivity state at close to physiological pH, serum, and Ca2+ levels
Source: PLoS One. 2026 Sep 9;21(9):e0357567. doi: 10.1371/journal.pone.0357567 (PMC13557354; doi:10.1371/journal.pone.0357567)
Supplement: Supplementary file 6 [file pone.0357567.s006.pdf]

S6 Fig. DLS profile of control media supplemented with Calcium ions at different pH

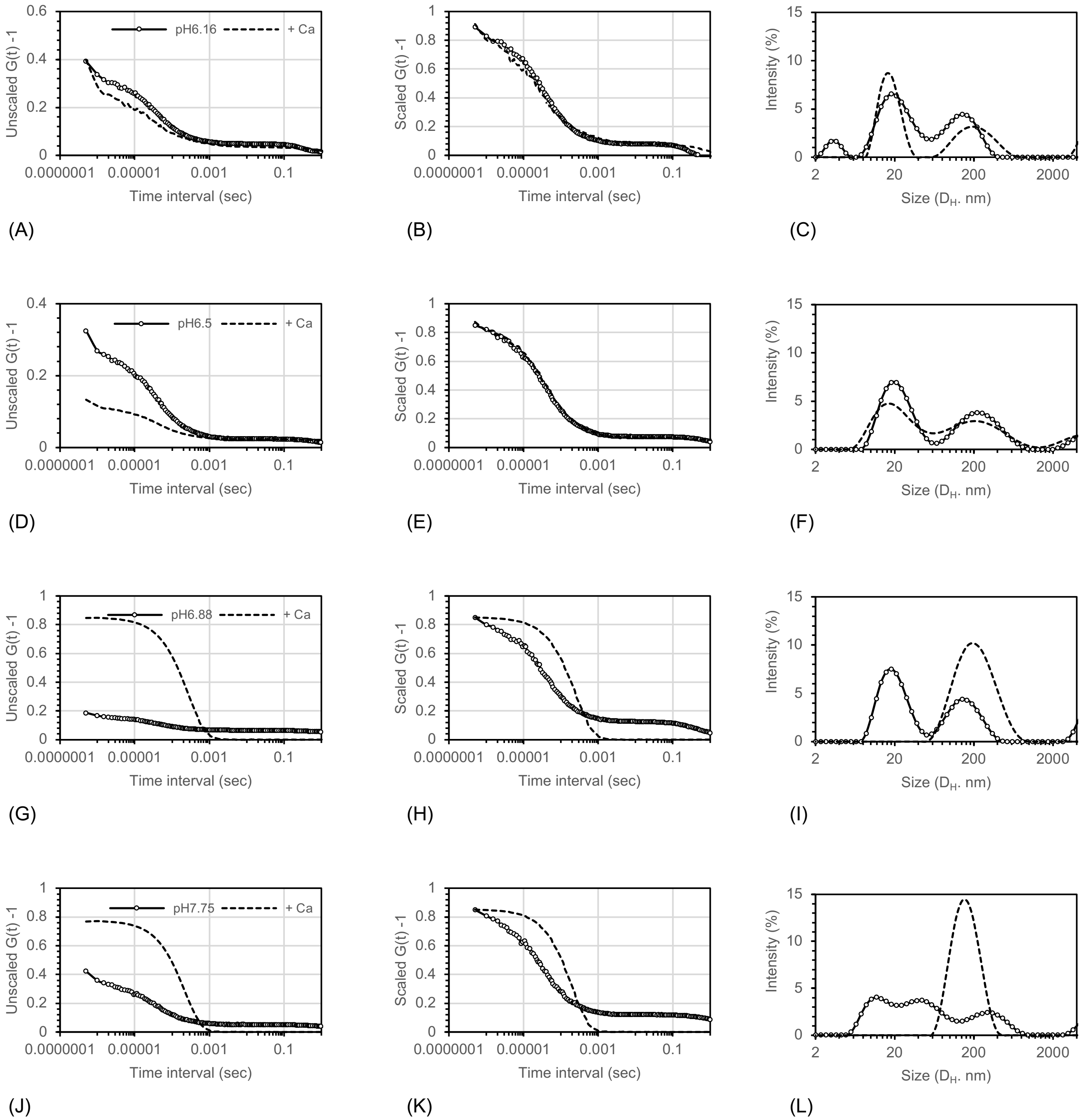

Control-media (diluted 10X in PBS) at various pH was supplemented with 4.2 mM Ca^{2+} ions. The unscaled and scaled correlation figures and D_H histograms are shown for pH: 6.16 (A-C); 6.5 (D-F); 6.88 (G – I); pH at 7.75 (J-L). The ~200 nm aggregate forms following Ca addition only for control media pH > 6.5.
