# Supplementary material for: HIV-1 lentivirus with VSV-G envelope: Complex N glycosylation governs shifts in aggregation and infectivity state at close to physiological pH, serum, and Ca2+ levels
Source: PLoS One. 2026 Sep 9;21(9):e0357567. doi: 10.1371/journal.pone.0357567 (PMC13557354; doi:10.1371/journal.pone.0357567)
Supplement: Supplementary file 7 [file pone.0357567.s007.pdf]

S7 Fig. Histogram of particle diameters observed in AFM imaging of the three states of the virus

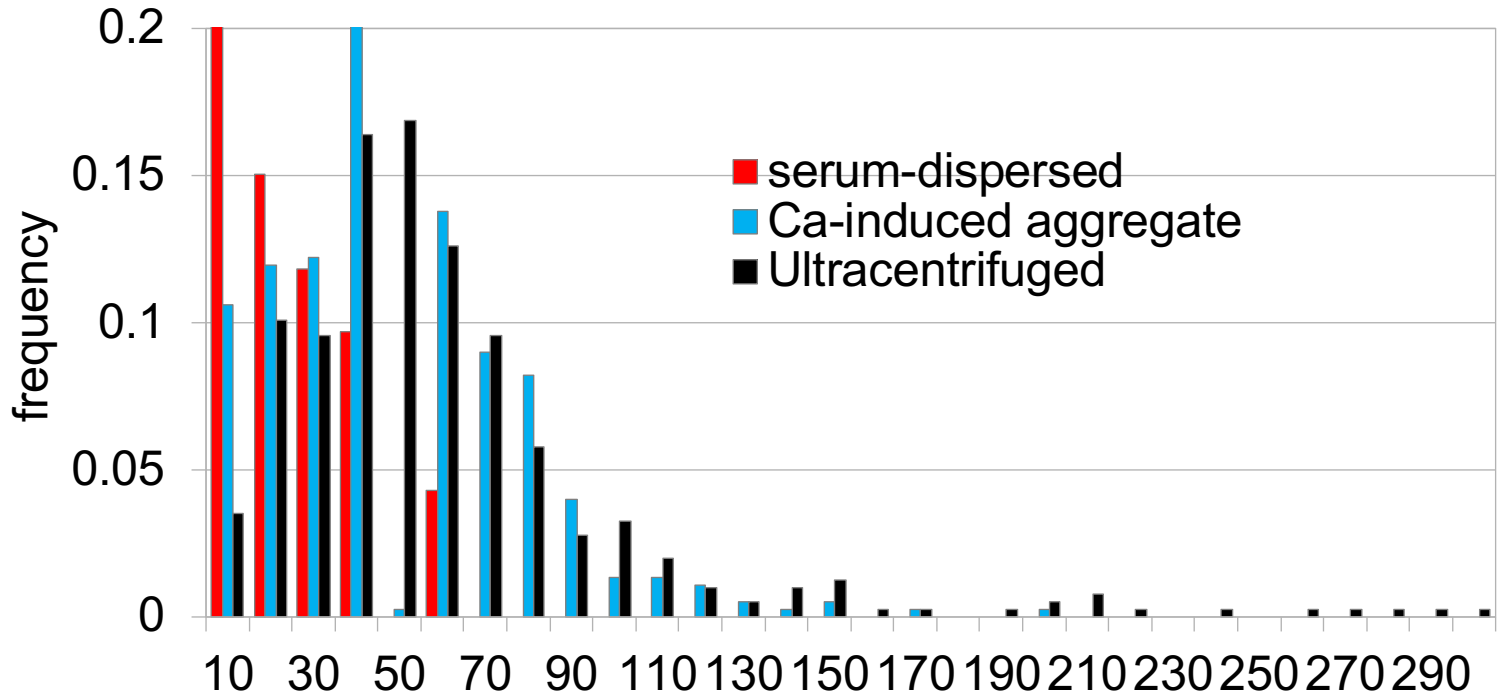

While there was a high preponderance of background particles observed in AFM, larger particles of virus aggregates were observed in Ca-induced and ultracentrifuged virus.
