# Supplementary material for: HIV-1 lentivirus with VSV-G envelope: Complex N glycosylation governs shifts in aggregation and infectivity state at close to physiological pH, serum, and Ca2+ levels
Source: PLoS One. 2026 Sep 9;21(9):e0357567. doi: 10.1371/journal.pone.0357567 (PMC13557354; doi:10.1371/journal.pone.0357567)
Supplement: Supplementary file 8 [file pone.0357567.s008.pdf]

S8 Fig. DLS profile of virus media following PNGase treatment

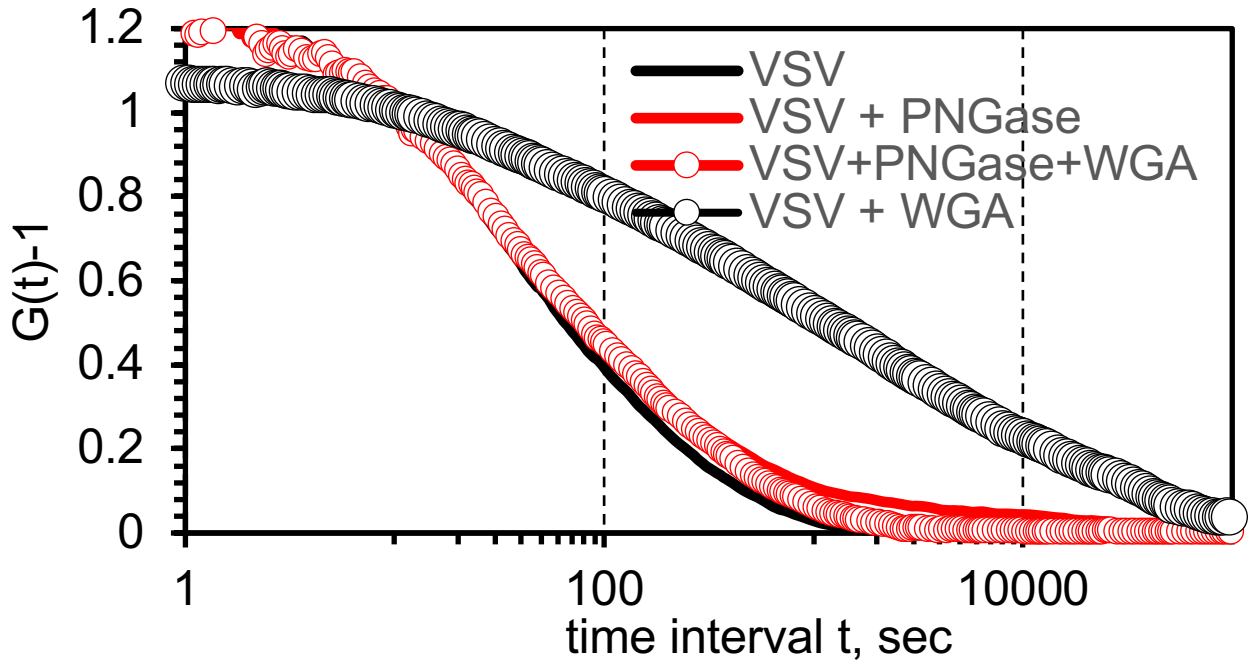

Correlation curves showing that virus solution (black) following PNGase treatment (red) ceases to show aggregation with WGA.
